# Breaking a dogma: acute anti-inflammatory treatment alters both post-lesional functional recovery and endogenous adaptive plasticity mechanisms in a rodent model of acute peripheral vestibulopathy

**DOI:** 10.1186/s12974-021-02222-y

**Published:** 2021-08-21

**Authors:** Nada El Mahmoudi, Guillaume Rastoldo, Emna Marouane, David Péricat, Isabelle Watabe, Alain Tonetto, Charlotte Hautefort, Christian Chabbert, Francesca Sargolini, Brahim Tighilet

**Affiliations:** 1 Aix-Marseille Université-CNRS, Laboratoire de Neurosciences Cognitives, LNC UMR 7291, Centre Saint Charles, Case C; 3 Place Victor Hugo, 13331 Marseille Cedex 03, France; 2 Centre Saint-Charles, Aix-Marseille Université CNRS, Case C; 3 Place Victor Hugo, 13331 Marseille Cedex 03, France; 3 Institut de Pharmacologie Et de Biologie Structurale, Université de Toulouse Paul Sabatier-CNRS, Toulouse, France; 4 Centrale Marseille, FSCM (FR 1739), PRATIM, Aix Marseille Université-CNRS, 13397 Marseille, France; 5 Department of Head and Neck Surgery, Lariboisière University Hospital, Paris, France; 6 GDR Physiopathologie Vestibulaire-Unité GDR2074 CNRS, Marseille, France

**Keywords:** Vestibular compensation, Inflammation, Corticosteroids, Acute peripheral vestibulopathies

## Abstract

**Background:**

Due to their anti-inflammatory action, corticosteroids are the reference treatment for brain injuries and many inflammatory diseases. However, the benefits of acute corticotherapy are now being questioned, particularly in the case of acute peripheral vestibulopathies (APV), characterized by a vestibular syndrome composed of sustained spinning vertigo, spontaneous ocular nystagmus and oscillopsia, perceptual-cognitive, posturo-locomotor, and vegetative disorders. We assessed the effectiveness of acute corticotherapy, and the functional role of acute inflammation observed after sudden unilateral vestibular loss.

**Methods:**

We used the rodent model of unilateral vestibular neurectomy, mimicking the syndrome observed in patients with APV. We treated the animals during the acute phase of the vestibular syndrome, either with placebo or methylprednisolone, an anti-inflammatory corticosteroid. At the cellular level, impacts of methylprednisolone on endogenous plasticity mechanisms were assessed through analysis of cell proliferation and survival, glial reactions, neuron’s membrane excitability, and stress marker. At the behavioral level, vestibular and posturo-locomotor functions’ recovery were assessed with appropriate qualitative and quantitative evaluations.

**Results:**

We observed that acute treatment with methylprednisolone significantly decreases glial reactions, cell proliferation and survival. In addition, stress and excitability markers were significantly impacted by the treatment. Besides, vestibular syndrome’s intensity was enhanced, and vestibular compensation delayed under acute methylprednisolone treatment.

**Conclusions:**

We show here, for the first time, that acute anti-inflammatory treatment alters the expression of the adaptive plasticity mechanisms in the deafferented vestibular nuclei and generates enhanced and prolonged vestibular and postural deficits. These results strongly suggest a beneficial role for acute endogenous neuroinflammation in vestibular compensation. They open the way to a change in dogma for the treatment and therapeutic management of vestibular patients.

## Introduction

Neuroinflammation is a cellular and molecular complex process, supporting the brain’s response to various aggressions such as injury, infection or stress. In the central nervous system (CNS), it systematically involves microglial cells, resident brain macrophages, and astrocytes [45, 46, 81]. Two types of inflammatory states must be distinguished, based on the intensity and duration of the insult [15, 88]. Acute neuroinflammation is the brain’s immediate response. As a transient, self-regulated reaction, it is thought to play a neuroprotective role by facilitating tissue repair and post-lesional recovery. Conversely, chronic inflammation is a self-propagating and long-lasting reaction caused by a persistent stress [92] or dysregulations of the acute inflammatory resolution process [88]. Chronic inflammation has deleterious consequences leading to neurodegeneration and is associated with CNS disorders [58, 90].

The harmful impact of chronic inflammation on brain tissues have led to the administration of anti-inflammatory compounds in patients from the acute phase. Due to their anti-inflammatory action, corticosteroids have been the reference treatment for brain injuries and many inflammatory diseases for many years [8, 30, 43, 66]. However, the benefits of this treatment are now questioned since it does not appear to improve patients’ recovery [43, 80].

This is also the case for patients suffering acute peripheral vestibulopathies (APV). APV is characterized by violent, debilitating rotatory vertigo, nystagmus, and cyclotorsion, during the acute phase [93, 96], along with various perceptual-cognitive, vegetative, and posturo-locomotor disorders constituting the so-called vestibular syndrome [9, 109]. Although the underlying cause has not yet been identified, the involvement of an inflammatory process has recently been proposed [47], consistent with the standard corticosteroid treatment [94, 95, 111]. However, it was recently shown that this therapeutic protocol does not significantly improve patients’ functional recovery [9, 32, 36, 85, 115]. This suggests that the acute neuroinflammation process may play an important role in vestibular post-lesional recovery.

**Fig. 1:**
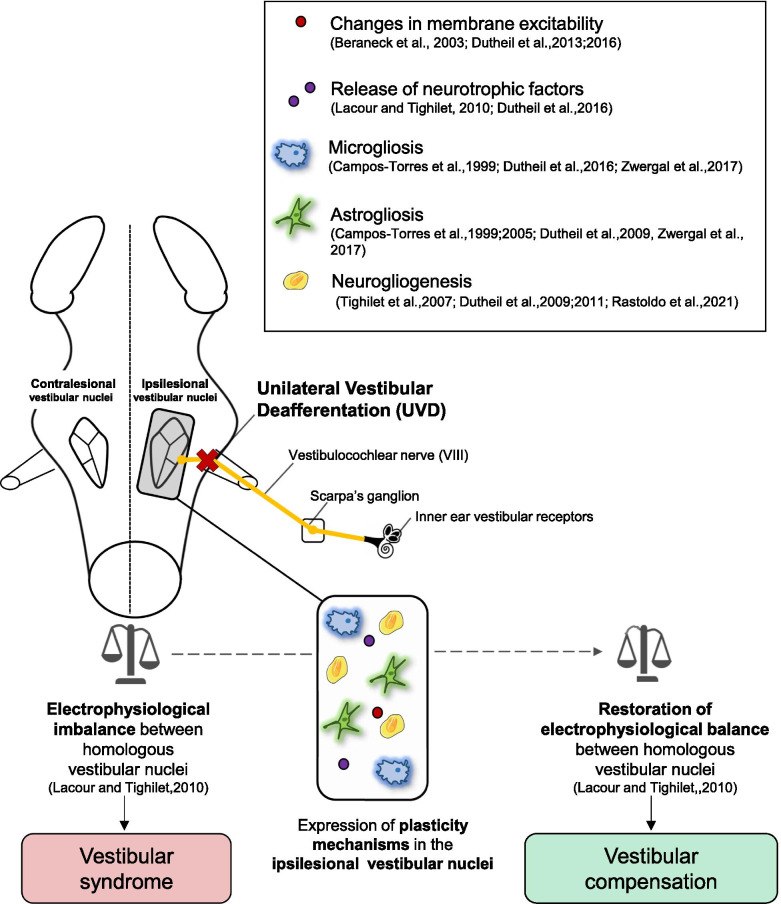
The vestibular compensation: a model a post-lesional neuroplasticity and functional recovery. Unilateral vestibular deafferentation (UDV) leads to a vestibular syndrome the origin of which is thought to be an electrophysiological imbalance between the ipsi- and contra-lesional vestibular nuclei (VNs). UVD leads to the emergence of a plethora of plasticity mechanisms in the ipsilesional VNs, supporting the progressive restoration of the electrophysiological balance and the subsequent functional recovery, called vestibular compensation

Among all unilateral vestibular deafferentation (UVD) models to study AVP, we focused on unilateral vestibular neurectomy (UVN), consisting in the section of one of the two vestibular nerves [50, 51, 70, 86]. UVN reproduces the human vestibular syndrome, which is thought to originate from an electrophysiological asymmetry between the ipsi- (weak activity) and contra-lesional (strong activity) vestibular nuclei (VNs) [26, 60, 71]. With time, the progressive and spontaneous restoration of the electrophysiological balance between the ipsi- and contra-lesional VNs supports the functional recovery that accompanies the disappearance of the vestibular syndrome [19, 49, 87]. This vestibular compensation is supported by the expression of several plasticity mechanisms in the deafferented vestibular environment (Fig. 1), such as changes in membrane excitability [6, 25], release of neurotrophic factors [25], and reactive neurogliogenesis [22, 74, 101].

UVN is also known to cause a neuroinflammatory reaction by inducing astroglial [22, 24, 75] and microglial [25, 75] responses, which are associated with the expression of two key inflammatory factors in the deafferented VNs: the tumor necrosis factor-alpha (TNF-alpha) and the nuclear factor-kappa B (NF-kB) [52]. UVN also activates the hypothalamo-pituitary-adrenal (HPA) axis [83, 106] leading to a strong release of anti-inflammatory endogenous corticosteroids (EC) in the deafferented VNs [106], thus confirming UVN-induced neuroinflammatory process.

The aim of this study is to assess the functional role of the acute neuroinflammation process in functional recovery after UVN. To do so, we investigated the effects induced by pharmacological blockade of acute inflammation following UVN on the expression of the plasticity mechanisms observed in the deafferented VNs, as well as on the kinetics of vestibular compensation in the adult rodent.

## Materials and methods

### Animals and ethical statements

This study was performed on 61 adult Long Evans female rats weighing between 250 and 350 g (10–12 weeks old at the beginning of the study). All experiments were performed in accordance with the National Institutes of Health’s Guide for Care and Use of Laboratory Animals (NIH Publication no. 80–23) revised in 1996 for the UK Animals (Scientific Procedures) Act of 1986 and associated guidelines or the Policy on Ethics approved by the Society for Neuroscience in November 1989 and amended in November 1993 and under veterinary and National Ethical Committee supervision (French Agriculture Ministry Authorization: B13-055–25). The present study was specifically approved by Neurosciences Ethics Committee N°71 of the French National Committee of animal experimentation. Every effort was made to minimize both the number and the suffering of animals used in this experiment. Rats had free access to food and water and were housed with a littermate in an enriched environment under a constant 12-h light.

### Study design

**Fig. 2:**
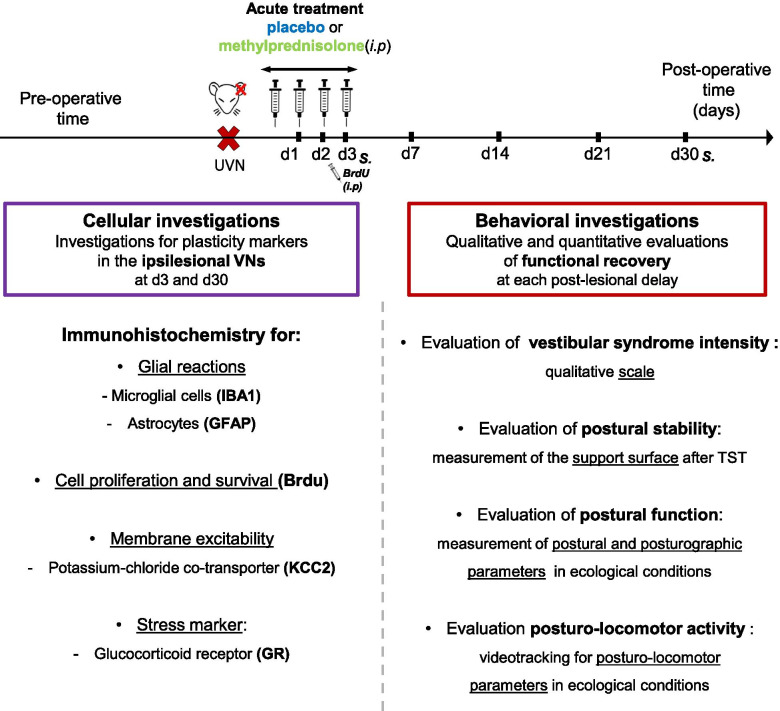
Study design. Experimental protocol to study and visualize the impact of the pharmacological blockage of the acute neuroinflammation after UVN on the expression of the plasticity mechanisms observed in the deafferented VN, as well as on the kinetics on functional recovery in the adult rat. *S* sacrifice*, i.p* intraperitoneal injections,. TST Tail Suspension Test

To determine the role of the acute inflammatory process in vestibular compensation, we used an anti-inflammatory compound, methylprednisolone (10 mg/kg), administrated intraperitoneally (i.p) immediately after the UVN and during the acute phase (first 3 days (d) after the lesion). We observed the effects of this treatment at both cellular and behavioral levels (Fig. 2). For that, we randomly divided the animals into 3 groups: a sham group (*n* = 21), submitted to the same surgical approach as UVN without sectioning the nerve; a UVN + placebo group (*n* = 21), lesioned and treated with NaCl 0.9%; and a UVN + methylprednisolone (UVN + met) group (*n* = 19), lesioned and treated with methylprednisolone. For each group, 4 animals were sacrificed at the end of the acute phase (d3) and 4 animals at the end of the behavioral study (d30) for cellular investigations. At the cellular level, we looked for changes in plasticity markers in the deafferented VNs at 3 and 30 days after the lesion. At the behavioral level, we measured the kinetics of the vestibular compensation using different behavioral assessments performed at different time points after the lesion.

### Unilateral vestibular neurectomy (UVN)

We used the model of left unilateral vestibular neurectomy in the adult rat [70] consisting in sectioning the left vestibular nerve. Animals were anesthetized with isoflurane (4%) 30 min after a subcutaneous injection of buprenorphine (Buprecare®,0.02 mg/kg). The animals were intubated, and the anesthesia was maintained during the surgery with isoflurane (3%). To access the left vestibular nerve, we used the tympanic bulla approach (see [70] for details) to access the vestibulocochlear nerve through the tympanic bulla and through the cochlea. The left vestibular nerve was then sectioned at a post-ganglion level, close to the brainstem. For the sham group (*n* = 22), the surgery was limited to the perforation of the tympanic bulla. Before awakening, the animals were injected subcutaneously with a solution of Ringer Lactate (Virbac; 10 ml/kg) to alleviate the dehydration resulting from the surgery. The success of the UVN (*n* = 44) was attested by the immediate appearance of a characteristic vestibular syndrome composed of postural, locomotor, and oculomotor deficits [70].

### Pharmacological treatments

The pharmacological treatments were administrated once per day during 3 days after the UVN, corresponding to the acute phase of the vestibular syndrome in rodents [70]. The UVN + placebo group was administrated with NaCl 0.9% (2 ml/kg) while the UVN + met group was treated with methylprednisolone (Solu-médrol®, 10 mg/kg), a corticosteroid classically used in vestibular patients [32, 36, 37, 95, 115].

### Cellular investigations

#### Tissue preparation

To study cell proliferation, animals received an i.p injection of 5-Bromo-2'-deoxyuridine (BrdU 200 mg/kg) dissolved in NaCl 0.9% 3 days after the lesion and were sacrificed either at 3 (*n* = 4 per group) or 30 days after the lesion (*n* = 4 per group) to assess short-term cell proliferation and long-term survival of the proliferative cells, respectively.

The rats were deeply anesthetized with a mixture of ketamine (Imalgène 1000®, 60 mg/kg) and medetomidine (Domitor® 0.25 mg/kg) for intracardiac perfusion. First, an intracardiac injection of 400 ml of isotonic saline (0.9% NaCl) was performed, followed by an injection of 400 ml of freshly prepared solution (4% paraformaldehyde (PFA) and in 0.1 M phosphate buffer (PB), pH 7.4). At the end of the perfusion, brains were extracted and post-fixed overnight at 4 °C in PFA 4% solution. Brains were then rinsed and cryoprotected by successive baths into sucrose solutions at increasing concentrations (10%, 20%, 30% of D-saccharose in 0.1 M PB each for 24 h at 4 °C). Brains were then frozen in dry ice and cut into serial 40 µm frontal sections with a cryostat (Leica) for immunochemistry.

#### Immunohistochemistry

Immunohistochemical labelling was performed according to previously validated protocols [23, 25, 75, 101]. Briefly, free-floating sections were first rinsed in 0.1 M phosphate-buffered saline (PBS) (3 × 5 min) before being saturated and permeabilized with a solution of bovine serum albumine (BSA) 5%; Triton X-100, 0.3% for 1 h. Incubation with primary antibody were performed in a solution of PBS 0.1 M; BSA 5%; Triton X-100 0.3% (24 h; 4 °C).

For BrdU immunohistochemistry, sections were incubated with a BrdU antibody (1:100, Dako, M0744). For glucocorticoid receptor (GR) immunohistochemistry, we used a GR antibody (1:300, Thermo Fisher, PA1-511A). For glial cells immunohistochemistry, we used a microglial marker, ionized calcium-binding adapter molecule 1 (IBA1) (1:2000, Wako, Cat#019–19,741), and an astrocytic marker, glial fibrillary acidic protein (GFAP) (1:200, Dako, Z033401-2). For potassium–chloride cotransporter 2 (KCC2) immunohistochemistry, sections were incubated with a KCC2 antibody (1:200, Merck, 07–432). For each section, we used DAPI (1:5000, Merck, D9542) incubation to mark the nucleus. Sections were then incubated in secondary antibodies dissolved in PBS 0.1 M for 1 h. We used goat anti-rabbit conjugated with Alexa Fluor 488 (1:500, Invitrogen, A11008) and goat anti-mouse IgG conjugated with Alexa Fluor 594 (1:500, Invitrogen, A11005) for immunostaining. Finally, sections were mounted with Roti®-mount fluorcare medium (Roth, HP19.1).

#### Cell counting methods and quantification of KCC2 immunoreactivity

Cell counts were performed according to previously validated protocols [22, 24, 25, 75]. All cellular investigations were performed in the left VNs at 3 and 30 days after left UVN or sham surgery. The recognition, localization and delimitation of the VNs of the brainstem was performed on cresyl violet-stained sections based on Paxinos and Watson’s stereotaxic atlas [67]. For quantification, 1 in 10 serial sections was used starting from the beginning (− 9.84 mm relative to the bregma) to the end of the VNs (− 13.08 mm relative to bregma [67]). Ten sequential sections of the deafferented (left) VNs were assessed. Immunoreactive (ir) cells were analyzed using confocal imaging with a Zeiss LM 710 NLO laser scanning microscope equipped with a 63X/1.32 BA oil immersion lens. Numbers of IBA1-, GFAP-, GR-, and BrdU-ir cells were counted using an integrated microscopic counting chamber that delineated the region of interest by a square of 425.10 mm^2^. The average cell counts from 10 ± 2 sections were used for statistical analysis. For GR quantification, we calculated the percentage of GR/DAPI ir-cells among GR-ir cells to assess for GR nuclear localization. For BrdU quantification, we calculated the percentage of BrdU ir-cells persisting at d30 to assess the survival of the proliferative cells observed at d3.

The quantification of the KCC2 immunolabeling was performed according to a previously published protocol [103] using a custom program written in MATLAB® (The Mathworks, Inc.). Briefly, the program allows analysis of the fluorescence at the plasma membrane of neurons. The background was assessed by calculating the average fluorescence in a visually selected area devoid of neurons or any other stained structure. From this region, a threshold was then derived, equal to the average immunofluorescence plus three times the standard deviation. All data were then subtracted from this threshold and only positive values were conserved for further analysis. A region of interest was drawn around the neuronal plasma membrane of each cell body. The program calculated the average fluorescence within the region of interest over data that were 20% above the maximum values. This thresholding insured that all pixels taken for calculating the average were part of the plasma membrane and that the same criterion was used for all slices in all conditions.

#### Cellular data statistical analysis

To avoid any bias, the cellular analysis was performed under blind conditions. For each cellular marker, the results are expressed as mean ± standard error mean (SEM). All analyses were performed with GraphPad Prism 9 (GraphPad Software, San Diego, CA). Normality of the data was controlled using the D’Agostino and Person normality test and by visualising quintile-quintile (Q-Q) plots. Since the normal distribution of the data was not rejected, we used parametric tests for statistical analyses and performed a two-way ANOVA to test the impact of the post-operative time and the impact of the group on the expression of the cellular markers, followed by post hoc Tukey’s multiple comparisons analysis. Results were considered significant at *p* < 0.05.

### Behavioral investigations

For each behavioral investigation, acquisitions were performed before the surgery (preop) and at different time-points (days) during the post-operative time (d1, d2, d3, d7, d14, d21, and d30) to assess, for each group, the intensity of the vestibular syndrome and the kinetics of the vestibular compensation.

#### Qualitative assessment of the vestibular syndrome

We assessed the intensity of the vestibular syndrome and its kinetic by using a cumulative qualitative scale listing typical postural, locomotor, and oculomotor deficits classically induced by UVN. Each behavioral symptom corresponds to a score on the qualitative scale (tumbling: 5; retropulsion: 4; circling: 3; bobbing: 2; head-tilt: 1). The score corresponds to the sum of the different symptoms, reflecting the severity of the vestibular syndrome and the alteration of the vestibular function (see [70] for details).

#### Support surface measurement after Tail Suspension Test (TST)

Vestibular function plays a crucial part in postural stability and posture-related responses according to behavioral context [61]. We assessed the postural stability after UVN by measuring the support surface (i.e., the area between the four paws of the animal), a well-known indicator used in various models of vestibular loss [25, 52, 59, 104]. To address directly the vestibular function, we performed the tail suspension test (TST) by holding the animal by the tail and subjecting it to vertical traction over a height of about 50 cm. Sudden vertical acceleration of the animal activates the remaining vestibular receptors, reactivating the vestibular syndrome while drastically reducing tactile and proprioceptive inputs [14, 107], leaving the effectiveness of the vestibulo-spinal reflex principally under the control of the vestibular function. The animals were placed in an open field and a picture was taken each time the animal landed after the TST test. The support surface was calculated in cm^2^, using an image analysis system developed on MATLAB®. For each animal, 10 repeated measurements were taken and averaged before the operation (preop) and at each post-operative time-point starting from d3. All the measurements were normalized so that each animal acted as its own control. To do so, for each rat, we used the preop value as the baseline (referenced as 1 for each rat) and we compared each value measured during the post-operative time to the baseline to visualize the changes of the support surface.

#### Quantitative assessment of postural function

We used the second version of the dynamic weight-bearing device (DWB2®) to assess) and quantify the postural function of rats after UVN, under ecological conditions (for details see [59, 107]. The animals were individually placed in this device and moved freely for 5 min. The apparatus allows quantification of the support forces of each part of the animal's body.

We analyzed the weight distribution of the animals along the lateral axis, previously described as a significant indicator of postural balance [59, 107]. It is represented in this study as the laterality index, corresponding to the difference in weight distributed between the right and left paws. We also used the DWB2® device to measure the rearing time (i.e., time spent on the two hind paws), considered as a significant indicator of the animal’s ability to stand, reflecting its postural balance control [107]. This apparatus enabled us to quantify a typical behavior of a unilateral vestibular loss, ipsilesional circling, defined as fast rotations of the animal toward the lesioned side [59, 70]. The circling behavior was assessed by counting the number of fast laps performed during an acquisition.

We also extracted posturographic parameters at the pre-operative time point and two post-operative time points: d3, corresponding to the acute phase of the vestibular syndrome, and d30, corresponding to the compensated phase of the vestibular syndrome. For the following parameters, data were normalized according to pre-operative values so that each rat acted as its own control:

- The average time spent by the animals with their abdomen on the ground sensors, during static and dynamic periods, which has been described and validated as a postural strategy during the acute phase after UVN [59]. This parameter, expressed in grams, was normalized according to pre-operative values by subtracting the pre-operative value to each post-lesional value measured during the post-operative time.
- The mean position of the barycenter which was calculated using the coordinates of each paw and their respective support forces (cf. equations (Eqs.) 3 and 4). The position of the barycenter was calculated during the acquisitions, at each time the animal was stationary and standing on its four paws.
  1 $$\mathrm{Barx}=\frac{\mathrm{FLx}*\mathrm{FLw }+\mathrm{ FRx}*\mathrm{FRw }+\mathrm{ RLx}*\mathrm{RLw }+\mathrm{ RRx}*\mathrm{RRw}}{\mathrm{FLw }+\mathrm{ FRw }+\mathrm{ RLw }+\mathrm{ RRw}}$$
  2 $$\mathrm{Bary}=\frac{\mathrm{FLy}*\mathrm{FLw }+\mathrm{ FRy}*\mathrm{FRw }+\mathrm{ RLy}*\mathrm{RLw }+\mathrm{ RRy}*\mathrm{RRw}}{\mathrm{FLw }+\mathrm{ FRw }+\mathrm{ RLw }+\mathrm{ RRw}}$$
  Based on the coordinates of the rat’s barycenter over time, we were able to trace the statokinesigram for each acquisition. Statokinesigrams show the trajectories in 2D of the barycenter and the center of gravity of each paw every time the calculation is performed, when rats were static and on their four paws. An average weighted by the duration of each of these moments is then established for each acquisition.
- We extracted the mean lateral position of the barycenter (in centimeters (cm)) that we normalized according to pre-operative values by subtracting the pre-operative value from each post-operative value measured during the post-operative time.
- To analyze the stability of the barycenter, we measured its maximum lateral deviation (maximum value of *Bary* minus minimum value of *Bary)* used here as an indicator of lateral instability. We also measured barycenter inertia, a measure of the barycenter positions’ dispersion during the acquisitions, reflecting postural stability. For both parameters, data were normalized as a ratio according to pre-operative values.

These acquisition methods have been recently published and validated for this rodent model of vestibulopathy by our group [59].

#### Quantitative assessment of the posturo-locomotor activity

We assessed the posturo-locomotor activity of the animals under ecological conditions by measuring different parameters known to be affected by UVN in rats (see [76] for details). At the beginning of the session, rats were placed individually in the center of an open field (80 × 80 × 40 cm) for 10 min and tracked with Ethovision ™ XT 14 software (Noldus). We measured the total distance moved (cm), the mean velocity (cm/s), and the mean acceleration (cm/s^2^) to assess the locomotor activity. These locomotor parameters were normalized by dividing each value measured during the post-operative time by the baseline (i.e., the value in the pre-operative time) to visualize the progression of the parameters. We also measured a postural variable, the mean body torsion defined as the angle between the nose and the tail of the rat during the acquisitions (expressed in degrees, positive values corresponding to torsion toward the left side and negative values to torsion toward the right side). This parameter was normalized for each rat by subtracting the baseline pre-operative value from the post-operative measures to visualize the increase or decrease of the parameter with time. These acquisition methods have been recently published and validated by our group [76].

#### Behavioral statistical analysis

The results are expressed as mean + SEM. All analyses were performed with GraphPad Prism 9 (GraphPad Software, San Diego, CA). Normality of the data was controlled using the D’Agostino and Person normality test and by visualizing quintile-quintile (Q-Q) plots. Since the normal distribution of the data was not rejected, we used parametric tests for statistical analyses, and performed a repeated measures ANOVA on 1-between (“group”), 1-within (“time”) design to test the impact of the post-operative time and the impact of the group on the behavioral markers, followed by post hoc analysis with Tukey’s multiple comparisons test. Results were considered significant at *p* < 0.05.

## Results

### Cellular results

#### Acute anti-inflammatory treatment significantly reduces glial reactions in the deafferented medial vestibular nuclei (VNs)

Glial reactions are crucial components of the inflammatory response in the CNS. To evaluate the impact of the acute anti-inflammatory treatment on glial responses, we used 2 well-known specific markers (IBA1 and GFAP).

**Fig. 3:**
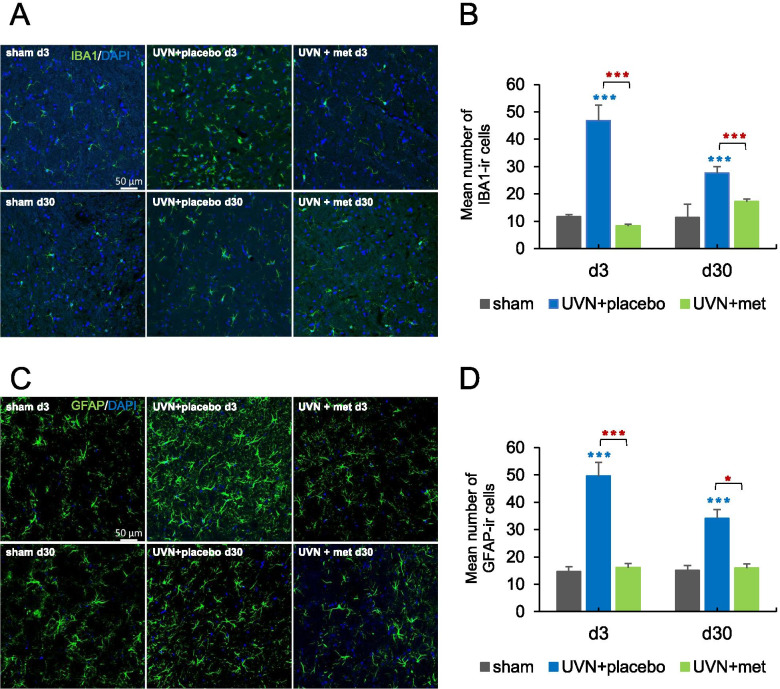
Glial reaction in the medial vestibular nuclei (VNs) are blocked by the acute anti-inflammatory treatment after UVN. **A** Confocal analysis of microglial cells immunostained with IBA1 and DAPI (nucleus) in the deafferented medial VNs of a representative animal in sham, UVN + placebo, and UVN + methylprednisolone (UVN + met) groups at d3 and d30 post-UVN. Scale bar, 50 mm. *N* = 4 animals per group. **B** Histograms showing the effects of vestibular lesion combined with placebo or acute anti-inflammatory treatment on the number of IBA1 immunoreactive cells in the deafferented medial VNs at d3 and d30 post-UVN. **C** Confocal analysis of astroglial cells immunostained with GFAP and DAPI (nucleus) in the deafferented medial VNs of a representative animal in sham, UVN + placebo, and UVN + met groups at d3 and d30 post-UVN. Scale bar, 50 mm. *N* = 4 animals per group. **D** Histograms showing the effects of vestibular lesion combined with placebo or acute anti-inflammatory treatment on the number of GFAP immunoreactive cells in the deafferented MVN at d3 and d30 post-UVN Each data point represents the mean number of immunoreactive cells, with error bars representing SEM. *p < 0.05; **p < 0.01; ***p < 0.001, 2 two-way ANOVA, post hoc Tukey: the sham and UVN + placebo comparison is indicated by a blue*; the sham and UVN + met comparison is indicated by a green*; and the UVN + placebo and UVN + met comparison is indicated by a red*

Immunohistochemistry investigation for the number of microglial cells using IBA1 antibody showed a significant decrease after UVN and acute anti-inflammatory treatment (Fig. 3A, B). Statistical analysis revealed a significant ‘group’ effect (two-way ANOVA; ‘group’: *F*(2,67) = 32.3, *p* < 0.001). In the sham group, we observed a basal and persistent number of IBA1-ir cells in the medial VNs. After UVN, there was a significant increase in the number of IBA1-ir cells in the deafferented medial VNs of the UVN + placebo group compared to the sham group at d3 (Tukey post hoc; *p* < 0.001), that persisted at d30 (Tukey post hoc; *p* < 0.05). Conversely, in the UVN + met group, no significant change was observed in the number of IBA1-ir cells compared to the sham group. Compared to the UVN + placebo group, the UVN + met group displayed a significantly lower number of IBA1-ir cells at d3 (Tukey post hoc; *p* < 0.001) and d30 (Tukey post hoc; *p* < 0.01).

Similarly, the number of astroglial cells, visualized with GFAP-ir cells, was reduced after UVN and acute anti-inflammatory treatment (Fig. 3C, D). The statistical analysis revealed significant ‘group’ ‘group × time’ effects (two-way ANOVA; ‘group’: *F*(2,63) = 43.1, *p* < 0.001; ‘group × time’: *F*(2,63) = 3.77, *p* < 0.05; ‘time’: *F*(1,63) = 2.87, *p* = 0.09). The quantification of the GFAP-ir cells in the sham group revealed a stable and moderate number of immunoreactive cells over time. After UVN, there was a significant increase in the number of GFAP-ir cells in the deafferented medial VNs of the UVN + placebo group compared to the sham group at d3 (Tukey post hoc; *p* < 0.001) persisting at d30 (Tukey post hoc; *p* < 0.01). As for IBA1-ir cells, we observed no significant changes in the number of GFAP-ir cells in the UVN + met group compared to the sham group. Compared with the UVN + placebo group, the UVN + met group showed a significant decrease in the number of GFAP-ir cells at d3 (Tukey post hoc; *p* < 0.001) persisting at d30 (Tukey post-hoc; *p* < 0.05).

Acute anti-inflammatory treatment significantly reduces GR nuclear localization in the medial VNs 3 days post-UVN

UVN is known to induce an activation of the stress axis leading to the release of endogenous corticosteroids acting on the glucocorticoid receptor (GR) [83, 106]. To evaluate the impact of the acute anti-inflammatory treatment, GR antibody was used as a stress-response marker.

**Fig. 4:**
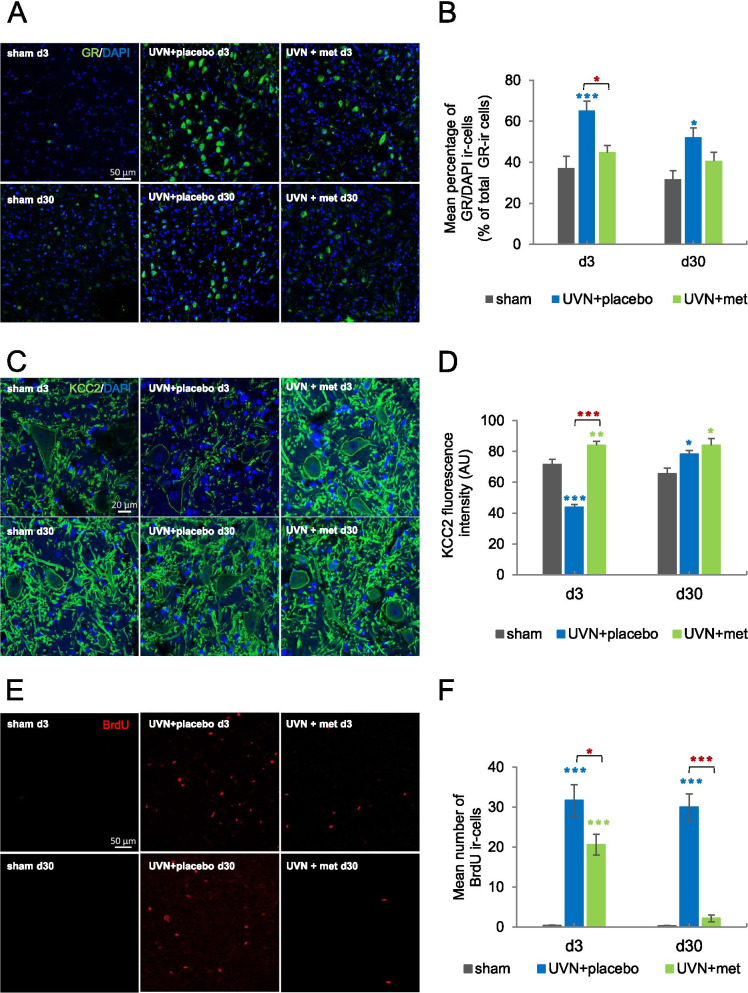
Glucocorticoid receptor (GR) localization, KCC2 expression, cell proliferation, and survival are altered by the acute anti-inflammatory treatment after UVN. **A** Confocal analysis of GR and nucleus (DAPI) localization in the deafferented medial VNs of a representative animal in sham, UVN + placebo, and UVN + met groups at d3 and d30 post-UVN. Scale bar, 50 mm. *N* = 4 animals per group. **D** Histograms showing the effects of vestibular lesion combined with placebo or acute anti-inflammatory treatment on the mean percentage of GR nuclear localization, quantified by the percentage of GR/DAPI ir-cells in the deafferented medial VNs at d3 and d30 post-UVN. **C** Confocal analysis of KCC2 expression in the deafferented medial VNs of a representative animal in sham, UVN + placebo, and UVN + met groups at d3 and d30 post-UVN. Scale bar, 20 mm. *N* = 4 animals per group. **D** Histograms showing the effects of the lesion combined with placebo or acute anti-inflammatory treatment on the mean KCC2 immunofluorescence intensity in the deafferented medial VNs at d3 and d30 post-UVN. **E** Confocal analysis of proliferative cells immunostained with BrdU in the deafferented medial VNs of a representative animal in sham, UVN + placebo, and UVN + met groups at d3 and d30 post-UVN. Scale bar, 50 mm. *N* = 4 animals per group. **F** Histograms showing the effects of the lesion combined with placebo or acute anti-inflammatory treatment on the number of Brdu immunoreactive cells in the deafferented medial VNs at d3 and d30 post-UVN. Each data point represents the mean number of immunoreactive cells, with error bars representing SEM. *p < 0.05; **p < 0.01; ***p < 0.001, 2 two-way ANOVA, post hoc Tukey: the sham and UVN + placebo comparison is indicated by a blue*; the sham and UVN + met comparison is indicated by a green*; and the UVN + placebo and UVN + met comparison is indicated by a red*

Immunohistochemistry for GR nuclear localization, investigated through the percentage of GR/DAPI ir-cells, revealed a significant decrease after acute anti-inflammatory treatment during the acute phase (Fig. 4A, B). Statistical analysis revealed a significant ‘group’ effect (two-way ANOVA; ‘group’: *F*(2,56) = 9.69, *p* < 0.001). For the sham group, the percentage of GR/DAPI ir-cells in the medial VNs was stable over time. After UVN and placebo treatment, strong nuclear GR immunoreactivity was attested by the significant increase of GR/DAPI at d3 compared to the sham group (Tukey post hoc; *p* < 0.01), persisting at d30 (Tukey post hoc; *p* < 0.05). Conversely, we did not observe in the UVN + met group any significant differences in the number of GR/DAPI ir-cells compared to the sham group. Compared to the UVN + placebo group, the UVN + met group showed a significant decrease in the number of GR/DAPI ir-cells at d3 (Tukey post hoc; *p* < 0.05).

#### Acute anti-inflammatory treatment alters proliferation and survival of new cells in the deafferented medial VNs

Different steps of neurogliogenesis (cell proliferation survival and differentiation) were shown to occur in the deafferented VNs after UVN [75] (rat model) [101], (feline model)) and to be crucial for vestibular compensation [22]. To assess the impact of the acute anti-inflammatory treatment on cell proliferation and survival, BrdU was used as a specific marker.

Investigations for cell proliferation and survival, analyzed using BrdU antibody, revealed a significant decrease of cell proliferation and survival after acute anti-inflammatory treatment (Fig. 4C, D). Statistical analysis revealed significant ‘time’, ‘group’ and ‘group × time’ effects (two-way ANOVA; ‘time’: *F*(1,45) = 6.95, *p* < 0.05; ‘group’: *F* (2,45) = 51.3, *p* < 0.001; ‘time × group’: *F* (2,45) = 5.48, *p* < 0.01).

In the sham group, we observed a very low rate of BrdU-ir cells. A strong and significant increase in the number of BrdU-ir cells was detected in the deafferented medial VNs for the UVN + placebo group compared to the sham group at d3 (Tukey post hoc; *p* < 0.001) and d30 (Tukey post hoc; *p* < 0.001), corresponding to 94.5% mean rate of survival. In the UVN + met group, we observed a significant increase of BrdU-ir cells at d3 compared to the sham group (Tukey post hoc; *p* < 0.05) but significantly lower compared to the UVN + placebo group (Tukey post-hoc; *p* < 0.05). In addition, in the UVN + met group, we observed, at d30, a significantly lower number of BrdU-ir cells compared to the UVN + placebo group (Tukey post hoc; *p* < 0.001) corresponding to a mean survival rate of 10.34%.

#### Acute anti-inflammatory treatment hinders the change in KCC2 expression in the lateral vestibular nuclei (VNs) 3 days post-UVN

Excitability level in the deafferented VNs is considered as a crucial parameter for vestibular compensation [102]. UVN was shown to modulate the expression of cation-chloride cotransporter KCC2 at neuron’s membrane [25] conditioning the action of GABA on the neuron membrane’s excitability [78].

We focused our analysis on the giant neurons of the lateral VNs as they contain excitatory glutamatergic neurons involved in vestibulo-spinal pathways. We observed a significant increase of KCC2 expression after UVN and acute anti-inflammatory treatment during the acute phase (Fig. 4E, F). Statistical analysis of KCC2 fluorescence intensity revealed significant ‘time’, ‘group’, and ‘group × time’ effects (two-way ANOVA; ‘time’: *F*(1,177) = 19.9, *p* < 0.001; ‘group’: *F*(3,177) = 59.9, *p* < 0.001; ‘time × group’: *F*(3,177) = 17.7, *p* < 0.001). For the sham group, a persistent mean of KCC2 fluorescence intensity was observed over time. A significant reduction in the mean KCC2 immunofluorescence intensity was observed in the UVN + placebo group compared to the sham group at d3 (Tukey post hoc; *p* < 0.001), no longer present 30 days after UVN. In the UVN + met group, a significant increase in the mean KCC2 fluorescence intensity was observed compared to the sham group at d3 (Tukey post hoc; *p* < 0.01) persisting at d30 (Tukey post hoc; *p* < 0.05). Compared to the UVN + placebo group, the UVN + met group displayed a significantly greater mean KCC2 fluorescence intensity at d3 (Tukey post hoc; *p* < 0.001).

### Behavioral results

#### Acute anti-inflammatory treatment significantly increases the intensity of the vestibular syndrome after UVN

**Fig. 5:**
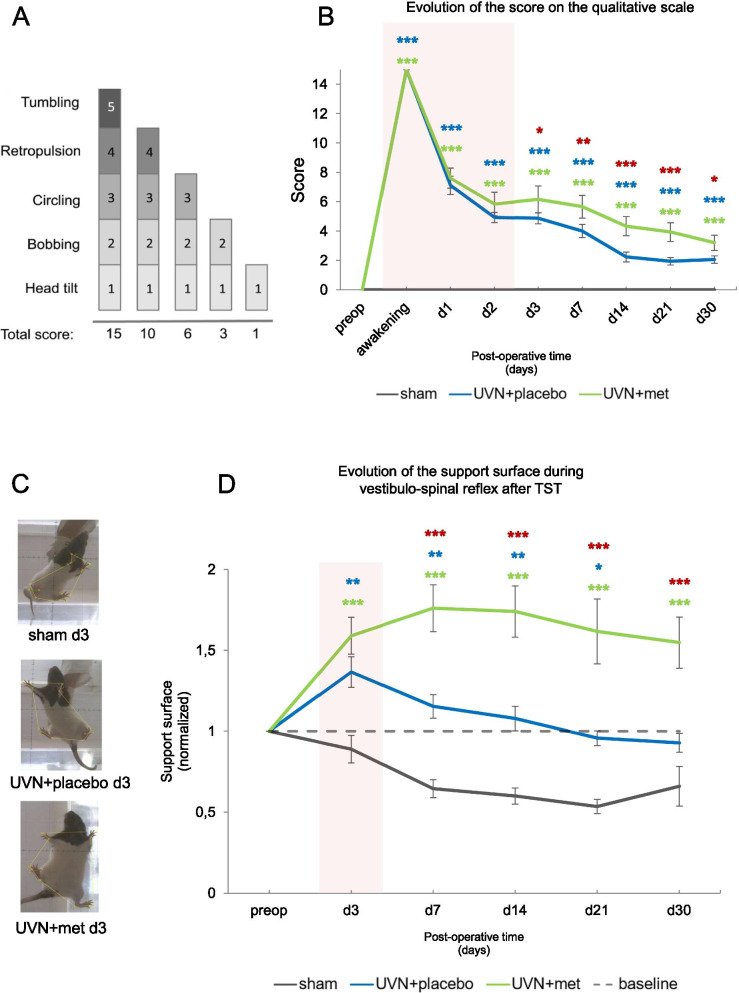
Acute anti-inflammatory after UVN treatment exacerbates vestibular syndrome intensity and postural instability during vestibulo-spinal reflex after UVN. **A** Illustration of the assessment grid used to conduct the qualitative analysis. **B** Results representing the progression of the qualitative score along post-operative time for each group. **C** Pictures of the support surface of a representative animal at d3 for each group. For each rat, a measurement was taken during the preoperative time to serve as baseline so that each rat acts as its own control. Data were normalized according to the baseline for every time-point. **D** Results representing the changes of the support surface measured after tail suspension test (TST), reflecting the effectiveness of the vestibulospinal reflex along post-operative time for each group. A red box is applied on the curve to illustrate the acute time window of the vestibular syndrome (d1 to d3) where the treatments were administrated daily. Each data point represents the mean for each group with error bars representing SEM. *p < 0.05; **p < 0.01; ***p < 0.001, 2 two-way ANOVA, post hoc Tukey: the sham and UVN + placebo comparison is indicated by a blue*; the sham and UVN + met comparison is indicated by a green*; and the UVN + placebo and UVN + met comparison is indicated by a red*

The intensity and time course of the vestibular syndrome were analyzed using a score on a qualitative scale, listing typical posturo-locomotor symptoms induced by UVN [70]. We observed a significantly increased vestibular syndrome after UVN and acute anti-inflammatory treatment (Fig. 5A, B). Statistical analysis revealed significant ‘time’, ‘group’, and ‘time × group’ effects (two-way repeated measures ANOVA, ‘time’: *F*(8,368) = 280, *p* < 0.001; ‘group’: *F*(2,46) = 150, *p* < 0.001; ‘time × group’: *F*(16,368) = 73.9, *p* < 0.001). We observed for the UVN + placebo group a characteristic kinetic pattern [59, 70, 107], with an intense vestibular syndrome during the first 3 days after UVN, decreasing progressively over time but still significantly persistent compared to the sham at all time points (Tukey post hoc,*p* < 0.001 at all-times). In the UVN + met group, the vestibular deficits were significantly greater compared to the UVN + placebo group at all time points from d3 (Tukey post hoc; *p* < 0.05) to d30 (Tukey post hoc; *p* < 0.05) indicating an intensified vestibular syndrome after acute anti-inflammatory treatment.

#### Acute anti-inflammatory treatment significantly increases postural instability during vestibulo-spinal reflex reactivation after UVN

To assess the postural stability after UVN, we analyzed the support surface after tail suspension test (TST). In four-footed animals, vestibular syndrome leads to an increased support surface delimited by the four paw pads [52, 59, 108]. This parameter provides a good estimation of postural instability after UVN since it displays the tonic asymmetry of extensor and flexor muscles of the anterior and posterior paws that results from vestibular deafferentation [105]. In addition, this parameter is measured after TST, which enables us to appreciate the effectiveness of the vestibulo-spinal reflex under the control of vestibular function recovery [14, 39, 107]. We observed a significant increase of postural instability after UVN and acute anti-inflammatory treatment (Fig. 5C). Statistical analysis revealed significant ‘time’, ‘group’, and ‘time × group’ effects (two-way repeated measures ANOVA, ‘time’: *F*(5,145) = 7.46, *p* < 0.001; ‘group’: *F*(2,29) = 24.5, *p* < 0.001; ‘time × group’: *F*(10,145) = 11.1, *p* < 0.001). For the sham group, we observed a significant diminution of the support surface over time (preop versus d30; Tukey post hoc; *p* < 0.05), probably reflecting habituation to the test. Conversely, a significant increase of the support surface was observed in the UVN + placebo group compared to the sham group at d3 (Tukey post hoc; *p* < 0.01). This significant difference persisted until d21 (Tukey post hoc; *p* < 0.05) and was no longer present at d30. A significant increase of the support surface parameter was also observed in the UVN + met group compared to the sham group from d3 (Tukey post hoc; *p* < 0.001) to d30 (Tukey post hoc; *p* < 0.001). The enlargement of the support surface was significantly more pronounced in the UVN + met group compared the UVN + placebo group from d7 (Tukey post hoc; *p* < 0.001) to d30 (Tukey post hoc; *p* < 0.001), indicating enhanced and persistent postural instability in the UVN + met group over time.

#### Effect of acute anti-inflammatory treatment on postural parameters analyzed by the dynamic weight distribution device

##### Weight distribution along the lateral axis

**Fig. 6:**
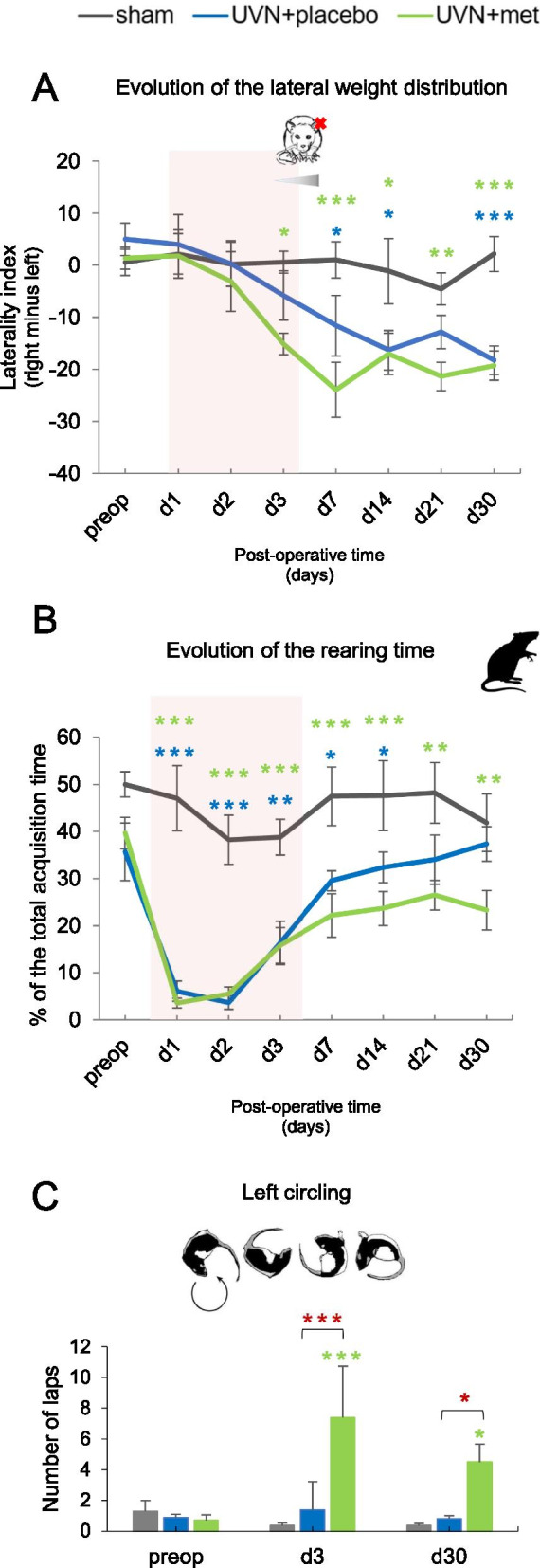
Acute anti-inflammatory treatment effects on weight distribution after UVN. **A** Results representing the changes of the lateral weight distribution index along post-operative time for each group. Positive values indicate an increase of the weight on the right paws, and negative values indicate an increase of the weight on the left paws. **B** Results representing the evolution of the rearing time (i.e., time spent on two paws) along post-operative time for each group. A red box is applied on the curve to illustrate the acute time window of the vestibular syndrome (d1 to d3) where the treatments were administrated daily. **C** Results representing the ipsilesional circling behavior (i.e., left circling). Each data point represents the mean for each group with error bars representing SEM. *p < 0.05; **p < 0.01; ***p < 0.001, 2 two-way ANOVA, post hoc Tukey: the sham and UVN + placebo comparison is indicated by a blue*; the sham and UVN + met comparison is indicated by a green*; and the UVN + placebo and UVN + met comparison is indicated by a red*

Weight distribution along the lateral axis was recently proved to be a good indicator of postural stability [59, 107]. We represented a weight laterality index corresponding to the weight distributed on the right paws minus the weight distributed on the left paws. We observed a significant increase of the weight distributed on the left paws after UVN for both UVN + placebo and UVN + met groups (Fig. 6A). Statistical analysis revealed significant ‘time’, ‘group’, and ‘time × group’ effects (two-way repeated measures ANOVA, ‘time’: *F*(7,140) = 10.8, *p* < 0.001; ‘group’: *F*(2,20) = 10.6, *p* < 0.001; ‘time × group’: *F*(14,140) = 2.39, *p* < 0.01). Similar to previous studies [59, 107], a significant increase of the weight distribution on the left paws was observed in the UVN + placebo group compared with the sham group at d7 (Tukey post hoc; *p* < 0.05), still present at d30 (Tukey post hoc; *p* < 0.01). In the UVN + met group, this increase appeared at d3 compared to the sham group (Tukey post hoc; *p* < 0.05) and increased over time until d30 (Tukey post hoc; *p* < 0.001).

##### Rearing time

The percentage of rearing time was used as an indicator of balance control, reflecting the animals' ability to stand [107]. We observed a significant decrease of the rearing time after UVN for both UVN + placebo and UVN + met groups (Fig. 6B). Statistical analysis revealed significant ‘time’, ‘group’, and ‘time × group’ effects (two-way repeated measures ANOVA, ‘time’: *F*(7,140) = 15.15, *p* < 0.001; ‘group’: *F* (2,20) = 35.5, *p* < 0.001; ‘time × group’: *F*(14,140) = 2.77, *p* < 0.01). For the sham group, we observed that the animals spent on average 50% of their time on the two hind paws. For the UVN + placebo group, we observed a significant decrease compared to the sham group at d1 (Tukey post hoc; *p* < 0.001) persisting until d14 (Tukey post hoc; *p* < 0.05). For the UVN + met group, we observed the same decrease compared to the sham group at d1 (Tukey post hoc; *p* < 0.001) persisting significantly until d30 (Tukey post hoc; *p* < 0.01). The rearing time was greater in the UVN + placebo group compared to the UVN + met group at d30 although not significantly (respectively 37.4% ± 3.7 versus 26.5% ± 5.2; Tukey post hoc; *p* = 0.05).

##### Left circling behavior (ipsilesional rotations)

We quantified the number of left circling (i.e., fast rotations toward the ipsilesional side), known to arise in UVN rat model during the acute phase [59, 70]. We observed a significant increase of the left circling after UVN and acute anti-inflammatory treatment (Fig. 6C). Statistical analysis revealed significant ‘time’, ‘group’, and ‘time × group’ effects (two-way repeated measures ANOVA, ‘time’: *F*(2,34) = 3.84, *p* < 0.05; ‘group’: *F*(2,17) = 10, *p* < 0.01; ‘time × group’: *F*(4,34) = 4.28, *p* < 0.01). In the UVN + placebo group, we observed that the left circling behavior was no longer present compared to the sham group, at both d3 and d30. In contrast, this behavior was significantly increased in the UVN + met group compared to the sham group at d3 (Tukey post hoc; *p* < 0.001) and d30 (Tukey post hoc; *p* < 0.05). Similar results were obtained when comparing the UVN + met and UVN + placebo groups with a significant increase of the left circling in the UVN + met group at d3 (Tukey post hoc; *p* < 0.001) and d30 (Tukey post hoc; *p* < 0.05).

##### Weight distributed on the abdomen

**Fig. 7:**
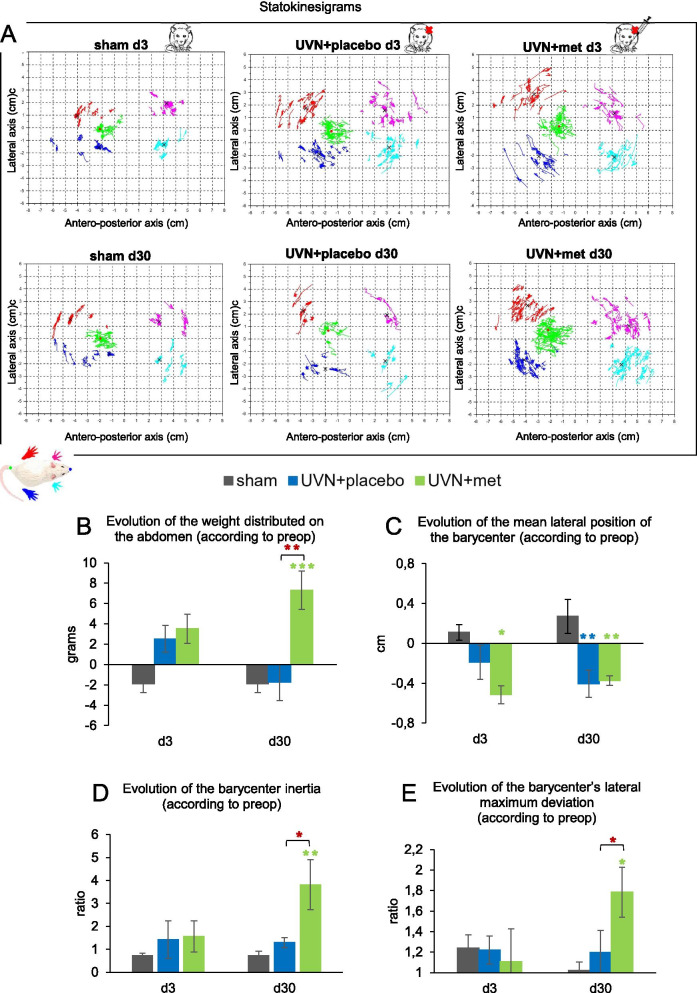
Acute anti-inflammatory treatment alters long-term postural recovery after UVN. **A** Statokinesigrams illustrating the kinetics of barycenter and paw positions at d3 and d30 for a representative animal in the sham, UVN + placebo and UVN + met groups. For each statokinesigram, the antero-posterior axis is on the abscissa and the lateral axis on the ordinate. The dark blue, red, light blue, and pink dot clouds are the traces of the average positions, respectively, of the right rear paws, left rear paws, right front, and left front paws during a session at each moment when the animal is static on its four legs. The green point cloud is the trace of the successive positions of the barycenter calculated at each of these moments. The various black crosses represent the average position of the legs during an entire session. The red dot represents the average position of the barycentre during a session. **B** Results representing the changes of the weight distributed on the abdomen, in grams, normalized by subtracting the preoperative value for each rat. **C** Results representing changes of the mean lateral position of the barycenter, in centimeters, normalized by subtracting the preoperative value for each rat. **D** Results representing changes of the barycenter inertia, an indicator of the barycenter stability. For each rat, measurements were normalized as a ratio of the preoperative value. **E** Results representing changes in the barycenter’s lateral maximum deviation, an indicator of barycenter stability along the lateral axis. For each rat, measurements were normalized as a ratio of the preoperative value. Each data point represents the mean for each group with error bars representing SEM. *p < 0.05; **p < 0.01; ***p < 0.001, 2 two-way ANOVA, post hoc Tukey: the sham and UVN + placebo comparison is indicated by a blue*; the sham and UVN + met comparison is indicated by a green*; and the UVN + placebo and UVN + met comparison is indicated by a red*

We quantified the weight distributed on the abdomen during the acquisitions. This parameter was shown to increase during the acute phase after UVN, especially during the first day [59], suggesting a strategy used by the animals to maintain balance with the use of a new support point to promote stability. We observed a significant increase of the weight distributed on the abdomen after UVN and acute anti-inflammatory treatment during the compensated phase (Fig. 7B). Statistical analysis revealed significant 'group' effect (two-way repeated measures ANOVA, ‘group’: *F*(2,17) = 10, *p* < 0.01). We observed that this postural strategy was absent in the UVN + placebo group compared to sham group at d3 and d30. In the UVN + met group, however, we observed that the animals distributed more weight on the abdomen at d30 compared to both sham (Tukey post hoc; *p* < 0.001) and UVN + placebo groups (Tukey post hoc; *p* < 0.01).

##### Barycenter posturographic analysis

Barycenter analysis was used to investigate posturographic parameters, similar to those used in clinic with vestibular patients [1, 59, 116].

We calculated the position of the barycenter each time the animal was stationary and standing on its four paws during the acquisitions. It enabled us to trace the positions of the paws and the average position of the barycenter for each animal, represented here as statokinesigrams (Fig. 7A). These statokinesigrams showed us different postural patterns depending on the group of rats and the post-lesion time, leading us to analyze 3 parameters representing the position and stability of the barycenter along over time.

##### Mean lateral position of the barycenter

We analyzed the patterns of change of the mean lateral position of the barycenter at d3 and d30. Positive values represent a displacement of the barycenter toward the right side while negative values represent a displacement of the barycenter toward the left side. We observed a significant displacement of the barycenter toward the left side after UVN for both UVN + placebo and UVN + met groups (Fig. 7C). Statistical analysis revealed significant 'group' effect (two-way repeated measures ANOVA, ‘group’: *F*(2,18) = 8.5, *p* < 0.01). In the UVN + placebo group, we observed a significant displacement of the barycenter toward the left side compared to the sham group at d30 (Tukey post hoc; *p* < 0.01). For the UVN + met group, this displacement toward the left side was significant at d3 compared to the sham group (Tukey post hoc; *p* < 0.01) and still present at d30 (Tukey post hoc; *p* < 0.01).

##### Barycenter inertia

We analyzed the barycenter inertia, a measure of the barycenter position dispersion during the acquisitions, reflecting its stability. We observed a significant increase of the barycenter inertia after UVN and acute anti-inflammatory treatment during the compensated phase (Fig. 7D). Statistical analysis revealed significant ‘group’ effect (two-way repeated measures ANOVA, ‘group’: *F*(2,19) = 5.33, *p* < 0.05). This parameter was significantly greater in the UVN + met group at d30 compared to both sham (Tukey post hoc; *p* < 0.01) and UVN + placebo groups (Tukey post hoc; *p* < 0.05).

##### Barycenter maximum lateral deviation

We analyzed the stability of the barycenter along the lateral axis by calculating the barycenter maximum lateral deviation (By_max_ minus By_min_). We observed a significant increase of the barycenter maximum lateral deviation after UVN and acute anti-inflammatory treatment during the compensated phase (Fig. 7E). Statistical analysis revealed significant ‘group × time’ effect (two-way repeated measures ANOVA, ‘group × time’: *F*(2,19) = 5.75, *p* < 0.05). This parameter was greater in the UVN + met group at d30 compared to sham (Tukey post hoc; *p* < 0.05) and UVN + placebo (Tukey post hoc; *p* < 0.05) groups.

#### Effect of acute anti-inflammatory treatment on posturo-locomotor parameters analyzed by videotracking

 New biomarkers of posturo-locomotor instability were recently identified by our group in the same UVN rat model [76]. We used the same analyses for the present study to investigate the impact of the acute anti-inflammatory treatment.

##### Mean body torsion of the animals

**Fig. 8:**
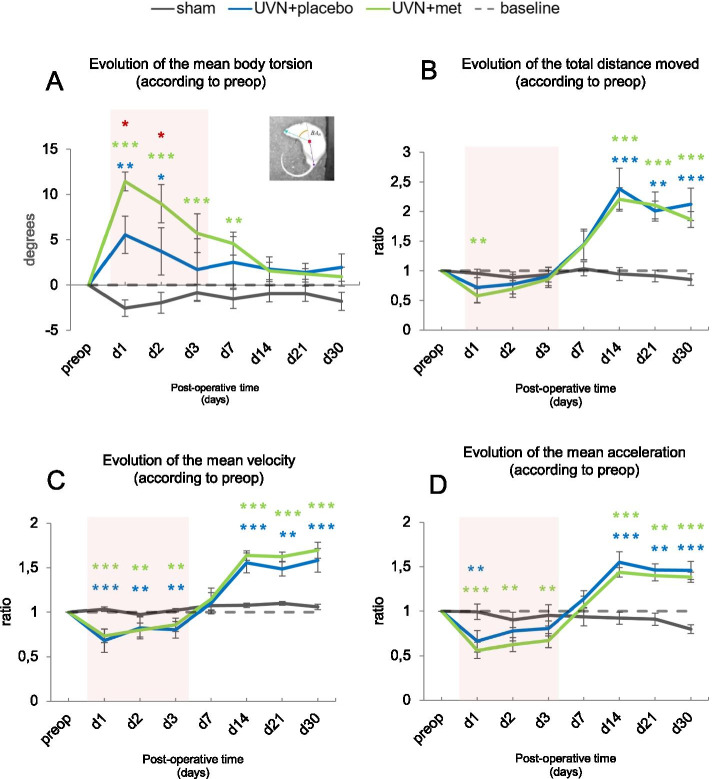
Acute anti-inflammatory treatment exacerbates postural alteration and has no beneficial effects on locomotion after UVN. **A** Results representing changes in the mean body torsion, in degrees, along post-operative time for each group, normalized by subtracting the pre-operative value for each rat. Positives values indicate an increase in body torsion toward the left side while negative values indicate an increase towards the right side. **B** Results representing changes in the total distance moved along post-operative time for each group, normalized as a ratio of the pre-operative value. **C** Results representing changes in the mean velocity along post-operative time for each group, normalized as a ratio of the pre-operative value. **D** Results representing changes in the mean acceleration along post-operative time for each group, normalized as a ratio of the pre-operative value. A red box is applied on the curve to illustrate the acute time window of the vestibular syndrome (d1 to d3) where the treatments were administrated daily. Each data point represents the mean for each group with error bars representing SEM. *p < 0.05; **p < 0.01; ***p < 0.001, 2 two-way ANOVA, post hoc Tukey: the sham and UVN + placebo comparison is indicated by a blue*; the sham and UVN + met comparison is indicated by a green*; and the UVN + placebo and UVN + met comparison is indicated by a red*

We measured the changes in the mean body torsion over time following UVN. This parameter has previously been shown to reflect postural alteration after unilateral vestibular deafferentation (UVD) [110]. Positive values represent increased body torsion towards the left side (side of the vestibular lesion) and negative values indicate increased body torsion towards the right side. We observed a significant increase of the mean body torsion towards the left side after UVN and acute anti-inflammatory treatment during the acute phase (Fig. 8A). Statistical analysis revealed significant ‘time’, ‘group’, and ‘time × group’ effects (two-way repeated measures ANOVA, ‘time’: *F*(7,217) = 6.83, *p* < 0.001; ‘group’: *F*(2,31) = 14.5, *p* < 0.001; ‘time × group’: *F*(14,217) = 6, *p* < 0.001). A significant increase of the mean body torsion toward the left side was observed for the UVN + placebo group compared with the sham group at d1 (Tukey post hoc; *p* < 0.001), persisting at d2 (Tukey post hoc, *p* < 0.05). This increase was significantly greater in the UVN + met group compared to the sham group at d1 (Tukey post hoc; *p* < 0.001) and persisted until d7 (Tukey post hoc; *p* < 0.05). It was significantly more pronounced in the UVN + met group compared to the UVN + placebo group at d1 (Tukey post hoc; *p* < 0.05) and d2 (Tukey post hoc; *p* < 0.05).

##### Total distance travelled

We investigated the total distance travelled in the open field. We observed a significant increase of this parameter after UVN for both UVN + placebo and UVN + met groups in the compensated phase (Fig. 8B). Statistical analysis revealed significant ‘time’, ‘group’, and ‘time × group’ effects (two-way repeated measures ANOVA, ‘time’: *F*(7,203) = 61.5, *p* < 0.001; ‘group’: *F*(2,29) = 4.94, *p* < 0.05; ‘time × group’: *F*(14,203) = 21.5, *p* < 0.001). We observed that the UVN + placebo group travelled a significantly greater distance than the sham group from d14 (Tukey post hoc; *p* < 0.001) until d30 (Tukey post hoc; *p* < 0.001). For the UVN + met group, we observed a significant decrease of the total distance travelled compared to the sham group at d1 (Tukey post hoc; *p* < 0.01), followed by a significant increase from d14 (Tukey post hoc; *p* < 0.001) to d30 (Tukey post hoc; *p* < 0.001).

##### Mean velocity during free locomotion

Regarding the mean velocity parameter, we observed a similar kinetics after UVN for both UVN + placebo and UVN + met groups (Fig. 8C). Statistical analysis revealed significant ‘time’ and ‘time × group’ effects (two-way repeated measures ANOVA, ‘time’: *F*(7,203) = 121, *p* < 0.001; ‘time × group’: *F*(14,203) = 32.4 *p* < 0.001). A significant decrease was observed in the UVN + placebo group compared to the sham group from d1 (Tukey post hoc; *p* < 0.001) to d3 (Tukey post hoc; *p* < 0.01). The opposite was observed from d14 to d30 with a significant increase of the mean velocity (Tukey post hoc; *p* < 0.001) persisting at d30 (Tukey post hoc; *p* < 0.001). Similar results were obtained in the UVN + met group with a significant decrease from d1 (Tukey post hoc; *p* < 0.001) to d3 (Tukey post hoc; *p* < 0.01) compared to the sham group. Again, the opposite was observed from d14 to d30 with a significant increase of the mean velocity (Tukey post hoc; *p* < 0.001) persisting at d30 (Tukey post hoc; *p* < 0.001).

##### Mean acceleration during free locomotion

Since vestibular receptors detect accelerations, this parameter is particularly relevant to assess vestibular dysfunction following UVN [76]. We observed a significant increase of this parameter after UVN for both UVN + placebo and UVN + met groups in the compensated phase (Fig. 8D). Statistical analysis revealed significant ‘time’ and ‘time × group’ effects (two-way repeated measures ANOVA, ‘time’: *F*(7,203) = 39.4, *p* < 0.001; ‘time × group’: *F*(14,203) = 17.3 *p* < 0.001). A significant decrease was observed in the UVN + placebo group compared to the sham group at d1 (Tukey post hoc; *p* < 0.05) followed by a significant increase from d14 (Tukey post hoc; *p* < 0.001) to d30 (Tukey post hoc; *p* < 0.001). For the UVN + met group, we also observed a significant diminution compared with the sham group at d1 (Tukey post hoc; *p* < 0.001) persisting until d3 (Tukey post hoc; *p* < 0.01), followed by a significant increase at d14 (Tukey post hoc; *p* < 0.001) persisting until d30 (Tukey post hoc; *p* < 0.001).

To resume the impact of the acute anti-inflammatory treatment on cellular and behavioral results, statistical comparisons between UVN + placebo and UVN + met groups were summarized in Table 1.

**Table 1:** Post hoc multiple comparisons for UVN + placebo and UVN + met groups based on Tukey’s procedure for cellular and behavioral analyses

Analysis	Delay	Mean UVN + placebo	Mean UVN + met	SE of diff	q	DF	*p* value*
Cellular analyses							
IBA1-ir cells	D3	46.8	8.33	5.86	9.28	67	< 0.0001***
	D30	36.1	13.9	7.24	4.34	67	0.0085***
GFAP-ir cells	D3	49.6	16.1	4.5	10.5	63	< 0.0001***
	D30	34.1	16	6.63	3.86	63	0.0219*
% GR/DAPI ir-cells	D3	65.3	44.8	8.08	3.58	56	0.0373*
BrdU-ir cells	D3	31.7	20.6	4.14	3.79	45	0.0271*
	D30	29.9	2.13	3.95	9.95	45	< 0.0001***
KCC2 fluorescence intensity	D3	43.8	84	3.96	14.3	162	< 0.0001***
Behavioral analyses							
Qualitative scale	D3	4.88	6.35	0.565	3.7	368	0.0251*
	D7	4	5.67	0.565	4.17	368	0.0095**
	D14	2.24	4.33	0.565	5.25	368	0.0007***
	D21	1.94	3.93	0.565	4.98	368	0.0014**
	D30	2.06	3.47	0.565	3.52	368	0.0352*
Support surface	D7	1.15	1.76	0.141	6.1	174	< 0.0001***
	D14	1.08	1.74	0.141	6.65	174	< 0.0001***
	D21	0.957	1.62	0.141	6.63	174	< 0.0001***
	D30	0.929	1.55	0.141	6.22	174	< 0.0001***
Left circling	D3	1.38	7.4	1.37	6.22	51	0.0002***
	D30	0.813	4.5	1.37	3.81	51	0.0255*
Weight distributed on the abdomen	D30	− 1.76	7.31	2.46	5.21	34	0.0022**
Evolution of the barycenter inertia	D30	1.29	3.81	0.868	4.1	38	0.0166*
Evolution of the barycenter’s lateral maximum deviation	D30	1.13	1.78	0.261	3.54	38	0.0432*
Mean body torsion	D1	5.56	11.5	2.1	3.97	248	0.0148*
	D2	3.73	8.99	2.1	3.55	248	0.0340*

Table summarizing the significant statistical differences observed between the UVN + placebo and the UVN + met groups for cellular and behavioral analyses during Tukey’s post hoc multiple comparisons test. *SE of diff* standard error for the difference between two means, *q q* ratio for Tukey’s post hoc multiple comparisons test, *p* probability level, *DF* degree of freedom, *p* < 0.05; **p < 0.01, ***p < 0.001

## Discussion

Due to their anti-inflammatory action, corticosteroids are the reference treatment for brain injuries and many inflammatory diseases, such as APV [94, 95, 111]. We used methylprednisolone, a corticosteroid, to assess the functional role of the endogenous acute neuroinflammation process in a rodent model of UVN. Here, we demonstrate that acute anti-inflammatory treatment has deleterious effects on vestibular compensation and disrupts the neuroplasticity mechanisms promoting functional recovery. Our results suggest for the first time a beneficial role of acute endogenous neuroinflammation in the expression of neuroplasticity mechanisms in the deafferented VN, promoting functional recovery after UVN.

### Acute anti-inflammatory treatment alters adaptive post-lesional plasticity in the deafferented VNs after UVN

Methylprednisolone is a synthetic corticosteroid, mimicking endogenous corticosteroids (EC) action on the glucocorticoid receptor (GR), an ubiquitous receptor expressed in almost all cells in mammals. When activated by a ligand, GR undergoes translocation into the nucleus to regulate the expression of genes encoding a variety of inflammatory proteins exerting anti-inflammatory and immunosuppressive actions [68, 77]. Interestingly, previous works reported the activation of the HPA axis after unilateral vestibular deafferentation (UVD) [10, 34, 106], leading to increased release of anti-inflammatory EC. Consistently, we observed an increased nuclear GR localization in the deafferented medial VNs after UVN. Although HPA axis activation after UVD is thought to be beneficial (for review, see [83], it has been reported that overexposure to corticosteroids has detrimental effects on vestibular compensation [113]. Following acute anti-inflammatory treatment, we observed a significant reduction of GR nuclear localization which is usually observed after desensitization of the receptor due to glucocorticoids overexposure [65]. The association of the acute anti-inflammatory treatment with endogenous corticosteroids (EC) probably leads to high concentrations of glucocorticoids in the deafferented VNs.

The restoration of the electrophysical balance between ipsi- and contra-lesional VNs, crucial for functional recovery, is supported at the cellular level by the expression of many neuroplasticity mechanisms in the deafferented VNs. We assessed the impact of acute anti-inflammatory treatment on post-lesional plasticity mechanisms by looking first at the glial responses, considered as hallmarks of the inflammatory response in the CNS. We observed that exposure to acute anti-inflammatory treatment significantly reduces astroglial and microglial reactions in the deafferented VNs after UVN. The expression of NF-kB, a key transcriptional factor for the inflammatory response [55, 72], is crucial for microglial and astroglial responses [53, 84]. GR activation is known to inhibit NF-kB so we can assume that GR activation, consecutive to the acute anti-inflammatory treatment combined with the release of endogenous corticosteroids, lead to the massive decreased glial responses observed in the deafferented VNs. Their inhibition after acute anti-inflammatory treatment is associated with altered vestibular compensation, probably highlighting their contribution to functional recovery. Glial reactions are crucial for adaptative post-lesional plasticity mechanisms in the VNs, since they promote preservation of tissue integrity and wound repair [15, 63, 73] and modulate neuronal network excitability through various mechanisms such as brain-derived-neurotrophic-factor (BDNF) signaling [16, 31].

Neurogenesis is an adaptive mechanism promoting vestibular compensation [22, 75]. We observed that acute anti-inflammatory treatment reduces cell proliferation and survival in deafferented medial VNs. Reduction of cell proliferation was also reported after overexposure to glucocorticoids in the hippocampus [2]. Altered neurogenesis is known to be associated with impaired functional recovery after UVN [22] and probably contributes to the exacerbated and persistent functional deficits observed after acute anti-inflammatory treatment. This may involve GR’s inhibition of NF-kB, which exerts a pro-proliferative effect on neural progenitors [112]. An alternative explanation might concern the inhibition of glial reactions, usually promoting neurogenesis through the release of BDNF [4, 27].

Finally, we examined the impact of acute anti-inflammatory treatment on vestibular neurons’ excitability in the deafferented VNs by focusing on GABAergic transmission. Our previous studies have shown that UVN induces a significant reduction of KCC2 expression in the deafferented lateral VNs [25], possibly leading to an intracellular accumulation of [Cl-] ions, inducing a depolarizing outward current through GABAA receptors [16, 17]. This mechanism likely plays a role in vestibular compensation through the transitory restoration of spontaneous activity in the deafferented VNs during the acute phase [25]. Here, we observed that acute anti-inflammatory treatment not only prevented KCC2 downregulation but significantly enhanced its expression in the deafferented lateral VNs. This phenomenon may induce an excitability deficit during the acute phase since KCC2 upregulation is likely associated with amplified inhibitory GABAergic transmission [7, 38, 56]. KCC2 upregulation was also reported after SCI and administration of corticosteroids [18]. The reactive microglia—BDNF—TrkB signaling was shown to be a main actor for KCC2 downregulation [16, 31, 78, 79]. Interestingly, KCC2 upregulation is thought to involve a TrkB receptor mediator, the Phospholipase C gamma (PLCy) [78, 99]. Previous works reported that glucocorticoids overexposure, as is likely the case after UVN and acute anti-inflammatory treatment, decreases PLCy binding to TrkB receptors [65] leading to KCC2 upregulation [78, 99].

In conclusion, we demonstrate that reactive plasticity mechanisms generated in the deafferented vestibular nuclei after UVN strongly depend on the acute inflammatory state, since their expression is prevented after acute anti-inflammatory treatment. Interestingly, it was reported that high doses of ibuprofen, a non-steroidal anti-inflammatory drug (NSAID), have deleterious consequences on hippocampal plasticity [35]. These results support the view that excessive inhibition of the inflammatory response impairs the expression of neural plasticity whatever the anti-inflammatory mechanism involved.

### Acute anti-inflammatory treatment after UVN alters the expression of the vestibular syndrome as well as the kinetics of vestibular compensation

To assess the consequences of acute anti-inflammatory treatment on functional recovery, we performed behavioral investigations to assess vestibular, postural, and locomotor functions [14, 59, 70, 76]. It is now widely accepted that acute vestibular syndrome originates from electrophysiological asymmetry between intact and deafferented VN and that recovery occurs through rebalance of electrical activity [102]. We used two behavioral tests that could assess the return to VNs electrophysiological homeostasis. First, we used a qualitative scale, listing typical postural and locomotor deficits classically induced by UVN and known to reflect vestibular function impairment and recovery [21, 70]. Then, we quantified the ipsilesional circling, a behavioral parameter observed in various rodent models of neuropathologies resulting from cerebral electrophysiological asymmetry [57, 91]. The acute administration of methylprednisolone exacerbates the severity of behavioral deficits in both tests. We can therefore assume that the acute anti-inflammatory treatment delays the return to electrophysiological homeostasis in the VNs and consequently, the vestibular compensation.

The VNs receive multimodal sensory inputs (vestibular, visual; tactile, and proprioceptive) and play a crucial role in postural stability, balance control, and reflex responses to body displacements through descending vestibulospinal pathways (see [61] for review). In the case of UVD, the loss of vestibular information from ipsilesional VNs and the subsequent VNs electrophysiological asymmetry leads to postural impairments (see [20] for review) that we assessed quantitatively. We observed, as previously described after UVN, an altered postural function during the acute phase as attested by the increase in the mean body torsion, the significant decrease of the rearing time and more unstable statokinesigrams compared to the sham group 3 days after the lesion. With time, we observed a progressive compensation of the postural parameters, concomitant with an increased weight distribution towards the injured side. This phenomenon was described as a compensatory mechanism, probably increasing tactile and proprioceptive inputs to the deafferented VNs, leading to a sensory reweighting. Increased sensitivity of vestibular neurons to proprioceptive inputs has been described after unilateral vestibular loss [44, 82] and is thought to support a sensory substitution mechanism, which is known to play a role in the vestibular compensation process (see [48] for review).

After acute methylprednisolone treatment, we observed enhanced short- and long-term postural deficits probably involving different plasticity mechanisms. We observed significantly enhanced body torsion toward the injured side during the acute phase but also increased instability of the barycenter during the compensated phase. These animals also exhibited significant and persistent use of the abdomen probably to improve postural balance through a somaesthetic substitution process. This hypothesis is supported by the measurement of the support surface after tail suspension test (TST), showing a strong impairment in UVN + met animals even 1 month after UVN, whereas UVN + placebo animals recovered over time. Under TST conditions, somaesthetic inputs are greatly reduced and the effectiveness of the vestibulo-spinal reflex is principally under the control of the recovery of vestibular function [14, 39]. We can argue that the long-term alteration of the plasticity mechanisms by acute methylprednisolone treatment causes the long-term alteration of the vestibulospinal reflex when mainly controlled by the vestibular function recovery.

Locomotor activity in rats after UVN was quantified in an automated and unbiased manner under ecological conditions, through the use of different quantitative parameters, recently validated as part of a specific posturo-locomotor phenotype after UVN [76]. In accordance with previous works, we observed a significant increase of these parameters with time, confirming the persistent hyperactivity after vestibular loss [54, 76]. This could represent a compensatory strategy since by increasing locomotion velocity, automatic spinal networks inhibit misleading vestibular information [29]. As previously described in rat models of spinal cord injury [40, 69, 114], acute anti-inflammatory treatment with methylprednisolone had no benefits for locomotion.

We argue that the acute treatment with methylprednisolone dysregulates the well-controlled endogenous balance between pro- and anti-inflammatory signals after UVN, leading to glucocorticoid overexposure. Acute methylprednisolone treatment alters both short- and long-term plasticity expression in the deafferented VNs, as well as enhanced and persistent vestibular and postural deficits. Interestingly, the inflammatory response was only blocked during the acute 3-day period after UVN but had both long-term cellular and behavioral consequences. Taken together, these results confirm the crucial role of this critical time period for functional recovery and highlight its potential therapeutic role.

### Clinical considerations

The UVN rodent model used in this work, displaying an acute phase of severe disorders, followed by a progressive reduction of the symptoms, faithfully mimics the vestibular syndrome encountered in most acute peripheral vestibulopathies (APV). Given its tissue correlation, it may be compared in first instance to vestibular neurotomy undertaken in the case of intractable Menière disease [62, 64] or vestibular schwannoma surgery [41]. In these two cases, central processes of vestibular neurons progressively degenerate through Wallerian degeneration, after being severed from their cell bodies located in the Scarpa’s ganglion. Based on the observation of inflammatory markers in the vestibular nuclei of UVN rats, it can be assumed that similar inflammation may take place following neurotomy and vestibular schwannoma. Present results are therefore of interest for the pharmacological management of patients in these conditions. Administration of corticoids within the appropriate time windows, avoiding the acute phase, may optimize functional recovery and stimulate vestibular compensation processes.

Although systemic inflammation has been described in vestibular neuritis patients [47], there is still no consistent evidence of a central inflammation in most cases [42, 109]. Rather, an intralabyrinthine source is now favored [28]. It can be assumed that vestibular primary neurons that compose the vestibular nerve remain alive although disconnected from the vestibular sensory cells [13, 100]. This situation differs slightly from the UVN model. However, among UVD models, chemical and surgical labyrinthectomy models have been reported to trigger inflammation and reactive plasticity mechanisms in the VNs [11, 12, 24, 52]. The presence of a central inflammatory reaction in vestibular neuritis should therefore be considered and the administration of acute corticosteroids should be questioned.

One might then ask why some clinical studies have reported significant benefits of acute corticotherapy in APV [3, 97]. It should be noted that the reported effectiveness of corticotherapy has mainly been based on the measurement of the vestibulo-ocular-reflex gain on caloric tests and did not include scales measuring the quality of life nor posturography measurements, better able to quantify central vestibular compensation. Furthermore, those results are now contested since meta-analysis has questioned the long-term benefits of acute corticotherapy [32, 36, 89], while recent studies proved no effectiveness of this treatment compared to vestibular re-education or other pharmacological treatment [37, 115].

The interest of understanding the inflammatory processes associated with vestibular pathologies extends well beyond the types of vestibular disorders mentioned above, as proinflammatory signatures have also been recently reported in Meniere disease and vestibular migraine [33]. In conclusion, this study using the UVN model raises new questions regarding the early use of systemic corticosteroids for the treatment of APV in humans. Further clinical studies will be necessary to validate the benefits of a reduction of their systematic use in human, while preferring other pharmacological or re-educational therapies.

## Conclusion

Our study strongly suggests, for the first time, that the pharmacological blockade of the acute inflammatory response after unilateral vestibular neurectomy alters the expression of the adaptative plasticity mechanisms in the ipsilesional VNs, involved in functional recovery. These results indicate that the endogenous acute neuroinflammation seems beneficial for vestibular compensation and question the use of corticosteroids in vestibular patients during the acute phase. The results also highlight a critical time window after the lesion since a treatment administrated during the acute phase has long-term effects.

## Abbreviations

APV: Acute peripheral vestibulopathies
CNS: Central nervous system
UVD: Unilateral vestibular deafferentation
UVN: Unilateral vestibular neurectomy
VNs: Vestibular nuclei
TNF-alpha: Tumor necrosis factor-alpha
NF-kB: Nuclear factor-kappa B
HPA: Hypothalamo-pituitary-adrenal
EC: Endogenous corticosteroids
i.p: Intraperitoneally
d: Days
met: Methylprednisolone
BrdU: 5-Bromo-2'-deoxyuridine
PB: Phosphate buffer
PFA: Paraformaldehyde
GR: Glucocorticoid receptor
IBA1: Ionized calcium-binding adapter molecule 1
KCC2: Potassium–chloride cotransporter 2
GFAP: Glial fibrillary acidic protein
SEM: Standard error mean
TST: Tail-Suspension Test
Equ: Equation
SCI: Spinal cord injury
BDNF: Brain-derived-neurotrophic-factor
PLCy: Phospholipase C gamma
NSAIDs: Non-steroidal anti-inflammatory drugs

## References

[1] Allum JH, Shepard NT (1999). An overview of the clinical use of dynamic posturography in the differential diagnosis of balance disorders. J Vestib Res.

[2] Anacker C, Cattaneo A, Luoni A, Musaelyan K, Zunszain PA, Milanesi E, Rybka J, Berry A, Cirulli F, Thuret S, Price J, Riva MA, Gennarelli M, Pariante CM (2013). Glucocorticoid-related molecular signaling pathways regulating hippocampal neurogenesis. Neuropsychopharmacol.

[3] Ariyasu L, Byl FM, Sprague MS, Adour KK (1990). The beneficial effect of methylprednisolone in acute vestibular vertigo. Arch Otolaryngol Head Neck Surg.

[4] Bath KG, Akins MR, Lee FS (2012). BDNF control of adult SVZ neurogenesis. Dev Psychobiol.

[5] Bellot-Saez A, Kékesi O, Morley JW, Buskila Y (2017). Astrocytic modulation of neuronal excitability through K+ spatial buffering. Neurosci Biobehav Rev.

[6] Beraneck M, Hachemaoui M, Idoux E, Ris L, Uno A, Godaux E, Vidal P-P, Moore LE, Vibert N (2003). Long-term plasticity of ipsilesional medial vestibular nucleus neurons after unilateral labyrinthectomy. J Neurophysiol.

[7] Bos R, Sadlaoud K, Boulenguez P, Buttigieg D, Liabeuf S, Brocard C, Haase G, Bras H, Vinay L (2013). Activation of 5-HT2A receptors upregulates the function of the neuronal K-Cl cotransporter KCC2. Proc Natl Acad Sci.

[8] Bracken MB, Collins WF, Freeman DF, Shepard MJ, Wagner FW, Silten RM, Hellenbrand KG, Ransohoff J, Hunt WE, Perot PL, Grossman RG, Green BA, Eisenberg HM, Rifkinson N, Goodman JH, Meagher JN, Fischer B, Clifton GL, Flamm ES, Rawe SE (1984). Efficacy of methylprednisolone in acute spinal cord injury. JAMA.

[9] Bronstein AM, Dieterich M (2019). Long-term clinical outcome in vestibular neuritis: current opinion in neurology.

[10] Cameron SA, Dutia MB (1999). Lesion-induced plasticity in rat vestibular nucleus neurones dependent on glucocorticoid receptor activation. J Physiol.

[11] Campos Torres A, Vidal PP, de Waele C (1999). Evidence for a microglial reaction within the vestibular and cochlear nuclei following inner ear lesion in the rat. Neuroscience.

[12] Campos-Torres A, Touret M, Vidal PP, Barnum S, de Waele C (2005). The differential response of astrocytes within the vestibular and cochlear nuclei following unilateral labyrinthectomy or vestibular afferent activity blockade by transtympanic tetrodotoxin injection in the rat. Neuroscience.

[13] Cassel, R., Bordiga, P., Carcaud, J., Simon, F., Beraneck, M., Gall, A.L., Benoit, A., Bouet, V., Philoxene, B., Besnard, S., Watabe, I., Pericat, D., Hautefort, C., Assie, A., Tonetto, A., Dyhrfjeld-Johnsen, J., Llorens, J., Tighilet, B., Chabbert, C., 2019. Morphological and functional correlates of vestibular synaptic deafferentation and repair in a mouse model of acute-onset vertigo. Disease Models & Mechanisms 12. 10.1242/dmm.039115 10.1242/dmm.039115 PMC6679379 31213478

[14] Cassel R, Bordiga P, Pericat D, Hautefort C, Tighilet B, Chabbert C (2018). New mouse model for inducing and evaluating unilateral vestibular deafferentation syndrome. J Neurosci Methods.

[15] Cherry JD, Olschowka JA, O’Banion M (2014). Neuroinflammation and M2 microglia: the good, the bad, and the inflamed. J Neuroinflammation.

[16] Coull JAM, Beggs S, Boudreau D, Boivin D, Tsuda M, Inoue K, Gravel C, Salter MW, De Koninck Y (2005). BDNF from microglia causes the shift in neuronal anion gradient underlying neuropathic pain. Nature.

[17] Coull JAM, Boudreau D, Bachand K, Prescott SA, Nault F, Sík A, Koninck PD, Koninck YD (2003). Trans-synaptic shift in anion gradient in spinal lamina I neurons as a mechanism of neuropathic pain. Nature.

[18] Dai S, Qi Y, Fu J, Li N, Zhang X, Zhang J, Zhang W, Xu H, Zhou H, Ma Z (2018). Dexmedetomidine attenuates persistent postsurgical pain by upregulating K^+^&ndash;Cl^&minus;^&nbsp;cotransporter-2 in the spinal dorsal horn in rats. JPR.

[19] Darlington CL, Smith PF (2000). Molecular mechanisms of recovery from vestibular damage in mammals: recent advances. Prog Neurobiol.

[20] Deliagina TG, Orlovsky GN, Zelenin PV, Beloozerova IN (2006). Neural bases of postural control. Physiology.

[21] Deliagina TG, Popova LB, Grant G (1997). The role of tonic vestibular input for postural control in rats. Arch Ital Biol.

[22] Dutheil S, Brezun JM, Leonard J, Lacour M, Tighilet B (2009). Neurogenesis and astrogenesis contribution to recovery of vestibular functions in the adult cat following unilateral vestibular neurectomy: cellular and behavioral evidence. Neuroscience.

[23] Dutheil S, Escoffier G, Gharbi A, Watabe I, Tighilet B (2013). GABA _A_ receptor agonist and antagonist alter vestibular compensation and different steps of reactive neurogenesis in deafferented vestibular nuclei of adult cats. J Neurosci.

[24] Dutheil S, Lacour M, Tighilet B (2011). Neurogenic potential of the vestibular nuclei and behavioural recovery time course in the adult cat are governed by the nature of the vestibular damage. PLoS ONE.

[25] Dutheil S, Watabe I, Sadlaoud K, Tonetto A, Tighilet B (2016). BDNF signaling promotes vestibular compensation by increasing neurogenesis and remodeling the expression of potassium-chloride cotransporter KCC2 and GABA _A_ receptor in the vestibular nuclei. J Neurosci.

[26] Dutia MB (2010). Mechanisms of vestibular compensation: recent advances: current opinion in otolaryngology & head and neck surgery.

[27] Ekdahl CT, Kokaia Z, Lindvall O (2009). Brain inflammation and adult neurogenesis: the dual role of microglia. Neuroscience, Brain - Immune Interactions in Acute and Chronic Brain Disorders.

[28] Eliezer M, Maquet C, Horion J, Gillibert A, Toupet M, Bolognini B, Magne N, Kahn L, Hautefort C, Attyé A (2019). Detection of intralabyrinthine abnormalities using post-contrast delayed 3D-FLAIR MRI sequences in patients with acute vestibular syndrome. Eur Radiol.

[29] Fabre-Adinolfi D, Parietti-Winkler C, Pierret J, Lassalle-Kinic B, Frère J (2018). You are better off running than walking revisited: Does an acute vestibular imbalance affect muscle synergies?. Hum Mov Sci.

[30] Fehlings MG, Wilson JR, Cho N (2014). Methylprednisolone for the Treatment of Acute Spinal Cord Injury: Counterpoint. Neurosurgery.

[31] Ferrini F, De Koninck Y (2013). Microglia control neuronal network excitability via BDNF signalling. Neural Plast.

[32] Fishman JM, Burgess C, Waddell A (2011). Corticosteroids for the treatment of idiopathic acute vestibular dysfunction (vestibular neuritis). Cochrane Database Syst Rev.

[33] Flook M, Frejo L, Gallego-Martinez A, Martin-Sanz E, Rossi-Izquierdo M, Amor-Dorado JC, Soto-Varela A, Santos-Perez S, Batuecas-Caletrio A, Espinosa-Sanchez JM, Pérez-Carpena P, Martinez-Martinez M, Aran I, Lopez-Escamez JA (2019). Differential proinflammatory signature in vestibular migraine and Meniere disease. Front Immunol.

[34] Gliddon CM, Darlington CL, Smith PF (2003). Activation of the hypothalamic–pituitary–adrenal axis following vestibular deafferentation in pigmented guinea pig. Brain Res.

[35] Golia, M.T., Poggini, S., Alboni, S., Garofalo, S., Ciano Albanese, N., Viglione, A., Ajmone-Cat, M.A., St-Pierre, A., Brunello, N., Limatola, C., Branchi, I., Maggi, L., 2019. Interplay between inflammation and neural plasticity: both immune activation and suppression impair LTP and BDNF expression. Brain, Behavior, and Immunity S0889159118312388. 10.1016/j.bbi.2019.07.003 10.1016/j.bbi.2019.07.003 31279682

[36] Goudakos JK, Markou KD, Franco-Vidal V, Vital V, Tsaligopoulos M, Darrouzet V (2010). Corticosteroids in the treatment of vestibular neuritis: a systematic review and meta-analysis.

[37] Goudakos JK, Markou KD, Psillas G, Vital V, Tsaligopoulos M (2014). Corticosteroids and vestibular exercises in vestibular neuritis: single-blind randomized clinical trial. JAMA Otolaryngol Head Neck Surg.

[38] Goulton, C.S., Watanabe, M., Cheung, D.L., Wang, K.W., Oba, T., Khoshaba, A., Lai, D., Inada, H., Eto, K., Nakamura, K., Power, J.M., Lewis, T.M., Housley, G.D., Wake, H., Nabekura, J., Moorhouse, A.J., 2018. Conditional Upregulation of KCC2 selectively enhances neuronal inhibition during seizures. bioRxiv 253831. 10.1101/253831

[39] Grosch M, Lindner M, Bartenstein P, Brandt T, Dieterich M, Ziegler S, Zwergal A (2021). Dynamic whole-brain metabolic connectivity during vestibular compensation in the rat. Neuroimage.

[40] Haghighi SS, Agrawal SK, Surdell D, Plambeck R, Agrawal S, Johnson GC, Walker A (2000). Effects of methylprednisolone and MK-801 on functional recovery after experimental chronic spinal cord injury. Spinal Cord.

[41] Halliday J, Rutherford SA, McCabe MG, Evans DG (2018). An update on the diagnosis and treatment of vestibular schwannoma. Expert Rev Neurother.

[42] Hegemann SCA, Wenzel A (2017). Diagnosis and treatment of vestibular neuritis/neuronitis or peripheral vestibulopathy (PVP)?. Open Questions and Possible Answers: Otology & Neurotology.

[43] Hurlbert RJ (2000). Methylprednisolone for acute spinal cord injury: an inappropriate standard of care. J Neurosurg Spine.

[44] Jamali M, Mitchell DE, Dale A, Carriot J, Sadeghi SG, Cullen KE (2014). Neuronal detection thresholds during vestibular compensation: contributions of response variability and sensory substitution: Neuronal detection thresholds during vestibular compensation. J Physiol.

[45] Jassam YN, Izzy S, Whalen M, McGavern DB, El Khoury J (2017). Neuroimmunology of traumatic brain injury: time for a paradigm shift. Neuron.

[46] Karve IP, Taylor JM, Crack PJ (2016). The contribution of astrocytes and microglia to traumatic brain injury: Neuroinflammation and TBI. Br J Pharmacol.

[47] Kassner SS, Schöttler S, Bonaterra GA, Stern-Straeter J, Hormann K, Kinscherf R, Gössler UR (2011). Proinflammatory activation of peripheral blood mononuclear cells in patients with vestibular neuritis. Audiology and Neurotology.

[48] Lacour M, Helmchen C, Vidal P-P (2016). Vestibular compensation: the neuro-otologist’s best friend. J Neurol.

[49] Lacour M, Tighilet B (2010). Plastic events in the vestibular nuclei during vestibular compensation: The brain orchestration of a &quot;deafferentation&quot; code. Restor Neurol Neurosci.

[50] Lacour M, Xerri C (1980). Compensation of postural reactions to free-fall in the vestibular neurectomized monkey. Exp Brain Res.

[51] Li H, Godfrey DA, Rubin AM (1995). Comparison of surgeries for removal of primary vestibular inputs: A combined anatomical and behavioral study in rats. Laryngoscope.

[52] Liberge, M., Manrique, C., Bernard-Demanze, L., Lacour, M., 2010. Changes in TNFa, NF B and MnSOD protein in the vestibular nuclei after unilateral vestibular deafferentation 16. 10.1186/1742-2094-7-91 PMC3004876 21143912

[53] Liddelow SA, Barres BA (2017). Reactive Astrocytes: Production, Function, and Therapeutic Potential. Immunity.

[54] Lindner M, Gosewisch A, Eilles E, Branner C, Krämer A, Oos R, Wolf E, Ziegler S, Bartenstein P, Brandt T, Dieterich M, Zwergal A (2019). Ginkgo biloba extract EGb 761 improves vestibular compensation and modulates cerebral vestibular networks in the rat. Front Neurol.

[55] Liu T, Zhang L, Joo D, Sun S-C (2017). NF-κB signaling in inflammation. Sig Transduct Target Ther.

[56] Lorenzo L-E, Godin AG, Ferrini F, Bachand K, Plasencia-Fernandez I, Labrecque S, Girard AA, Boudreau D, Kianicka I, Gagnon M, Doyon N, Ribeiro-da-Silva A, De Koninck Y (2020). Enhancing neuronal chloride extrusion rescues α2/α3 GABA A -mediated analgesia in neuropathic pain. Nat Commun.

[57] Löscher W (2010). Abnormal circling behavior in rat mutants and its relevance to model specific brain dysfunctions. Neurosci Biobehav Rev.

[58] Lucas S-M, Rothwell NJ, Gibson RM (2006). The role of inflammation in CNS injury and disease. Br J Pharmacol.

[59] Marouane, E., Rastoldo, G., El Mahmoudi, N., Péricat, D., Chabbert, C., Artzner, V., Tighilet, B., 2020. Identification of new biomarkers of posturo-locomotor instability in a rodent model of vestibular pathology. Front Neurol 11. 10.3389/fneur.2020.00470 10.3389/fneur.2020.00470 PMC7273747 32547480

[60] Mccabe BF, Ryu JH (1969). Experiments on vestibular compensation. Laryngoscope.

[61] McCall, A.A., Miller, D.M., Yates, B.J., 2017. Descending influences on vestibulospinal and vestibulosympathetic reflexes. Front. Neurol. 8. 10.3389/fneur.2017.00112 10.3389/fneur.2017.00112 PMC5366978 28396651

[62] Miyazaki H, Nomura Y, Mardassi A, Deveze A, Miura M, Jike M, Magnan J (2017). How minimally invasive vestibular neurotomy for incapacitating Meniere’s disease improves dizziness and anxiety. Acta Otolaryngol.

[63] Myer DJ (2006). Essential protective roles of reactive astrocytes in traumatic brain injury. Brain.

[64] Nevoux, J., Franco-Vidal, V., Bouccara, D., Parietti-Winkler, C., Uziel, A., Chays, A., Dubernard, X., Couloigner, V., Darrouzet, V., Mom, T., Groupe de Travail de la SFORL (2017). Diagnostic and therapeutic strategy in Menière’s disease. Guidelines of the French Otorhinolaryngology-Head and Neck Surgery Society (SFORL). Eur Ann Otorhinolaryngol Head Neck Dis.

[65] Numakawa T, Kumamaru E, Adachi N, Yagasaki Y, Izumi A, Kunugi H (2009). Glucocorticoid receptor interaction with TrkB promotes BDNF-triggered PLC- signaling for glutamate release via a glutamate transporter. Proc Natl Acad Sci.

[66] Paragliola RM, Papi G, Pontecorvi A, Corsello SM (2017). Treatment with synthetic glucocorticoids and the hypothalamus-pituitary-adrenal axis. IJMS.

[67] Paxinos G, Watson C (2009). The rat brain in stereotaxic coordinates.

[68] Payne DNR, Adcock IM (2001). Molecular mechanisms of corticosteroid actions. Paediatr Respir Rev.

[69] Pereira JE, Costa LM, Cabrita AM, Couto PA, Filipe VM, Magalhães LG, Fornaro M, Di Scipio F, Geuna S, Maurício AC, Varejão ASP (2009). Methylprednisolone fails to improve functional and histological outcome following spinal cord injury in rats. Exp Neurol.

[70] Péricat D, Farina A, Agavnian-Couquiaud E, Chabbert C, Tighilet B (2017). Complete and irreversible unilateral vestibular loss: A novel rat model of vestibular pathology. J Neurosci Methods.

[71] Precht W, Shimazu H, Markham CH (1966). A mechanism of central compensation of vestibular function following hemilabyrinthectomy. J Neurophysiol.

[72] Quax RA, Manenschijn L, Koper JW, Hazes JM, Lamberts SWJ, van Rossum EFC, Feelders RA (2013). Glucocorticoid sensitivity in health and disease. Nat Rev Endocrinol.

[73] Quintana, F.J., 2017. Astrocytes to the rescue! Glia limitans astrocytic endfeet control CNS inflammation. Journal of Clinical Investigation 127, 2897–2899.10.1172/JCI95769 10.1172/JCI95769 PMC5531401 28737511

[74] Rastoldo, G., 2021. Adult and endemic neurogenesis in the vestibular nuclei after unilateral vestibular neurectomy. Progress in Neurobiology 11. 10.1016/j.pneurobio.2020.101899 32858093

[75] Rastoldo G, El Mahmoudi N, Marouane E, Pericat D, Watabe I, Toneto A, López-Juárez A, Chabbert C, Tighilet B (2021). Adult and endemic neurogenesis in the vestibular nuclei after unilateral vestibular neurectomy. Prog Neurobiol.

[76] Rastoldo, G., Marouane, E., El Mahmoudi, N., Péricat, D., Bourdet, A., Timon-David, E., Dumas, O., Chabbert, C., Tighilet, B., 2020. Quantitative evaluation of a new posturo-locomotor phenotype in a rodent model of acute unilateral vestibulopathy. Front. Neurol. 11.10.3389/fneur.2020.00505 10.3389/fneur.2020.00505 PMC7291375 32582016

[77] Rhen T, Cidlowski JA (2005). Antiinflammatory action of glucocorticoids — new mechanisms for old drugs. N Engl J Med.

[78] Rivera C (2004). Mechanism of activity-dependent downregulation of the neuron-specific K-Cl cotransporter KCC2. J Neurosci.

[79] Rivera C, Li H, Thomas-Crusells J, Lahtinen H, Viitanen T, Nanobashvili A, Kokaia Z, Airaksinen MS, Voipio J, Kaila K, Saarma M (2002). BDNF-induced TrkB activation down-regulates the K+–Cl− cotransporter KCC2 and impairs neuronal Cl− extrusion. J Cell Biol.

[80] Russo MV, McGavern DB (2016). Inflammatory neuroprotection following traumatic brain injury. Science.

[81] Russo MV, McGavern DB (2015). Immune Surveillance of the CNS following Infection and Injury. Trends Immunol.

[82] Sadeghi SG, Minor LB, Cullen KE (2011). Multimodal integration after unilateral labyrinthine lesion: single vestibular nuclei neuron responses and implications for postural compensation. J Neurophysiol.

[83] Saman, Y., Bamiou, D.E., Gleeson, M., Dutia, M.B., 2012. Interactions between Stress and Vestibular Compensation – A Review. Front. Neur. 3. 10.3389/fneur.2012.00116 10.3389/fneur.2012.00116 PMC3406321 22866048

[84] Santa-Cecília FV, Socias B, Ouidja MO, Sepulveda-Diaz JE, Acuña L, Silva RL, Michel PP, Del-Bel E, Cunha TM, Raisman-Vozari R (2016). Doxycycline suppresses microglial activation by inhibiting the p38 MAPK and NF-kB signaling pathways. Neurotox Res.

[85] Shupak A, Issa A, Golz A, Kaminer M, Braverman I (2008). Prednisone treatment for vestibular neuritis. Otol Neurotol.

[86] Simon F, Pericat D, Djian C, Fricker D, Denoyelle F, Beraneck M (2020). Surgical techniques and functional evaluation for vestibular lesions in the mouse: unilateral labyrinthectomy (UL) and unilateral vestibular neurectomy (UVN). J Neurol.

[87] Smith PF, Curthoys IS (1989). Mechanisms of recovery following unilateral labyrinthectomy: a review. Brain Res Rev.

[88] Sochocka M, Diniz BS, Leszek J (2017). Inflammatory response in the CNS: friend or foe?. Mol Neurobiol.

[89] Solis RN, Sun DQ, Tatro E, Hansen MR (2019). Do steroids improve recovery in vestibular neuritis?: Steroids for Vestibular Neuritis. Laryngoscope.

[90] Stephenson J, Nutma E, van der Valk P, Amor S (2018). Inflammation in CNS neurodegenerative diseases Immunology.

[91] Stiles L, Smith PF (2015). The vestibular–basal ganglia connection: Balancing motor control. Brain Res.

[92] Streit WJ, Mrak RE, Griffin WST (2004). No title found. J Neuroinflammation.

[93] Strupp M, Brandt T (2009). Vestibular Neuritis. Semin Neurol.

[94] Strupp M, Brandt T (2009). Review: Current treatment of vestibular, ocular motor disorders and nystagmus. Ther Adv Neurol Disord.

[95] Strupp M, Dieterich M, Brandt T (2013). The treatment and natural course of peripheral and central vertigo. Deutsches Aerzteblatt Online.

[96] Strupp M, Mandalà M, López-Escámez JA (2019). Peripheral vestibular disorders: an update. Curr Opin Neurol.

[97] Strupp M, Zingler VC, Arbusow V, Niklas D, Maag KP, Dieterich M, Bense S, Theil D, Jahn K, Brandt T (2004). Methylprednisolone, valacyclovir, or the combination for vestibular neuritis. N Engl J Med.

[98] Takeda K, Sawamura S, Sekiyama H, Tamai H, Hanaoka K (2004). Effect of methylprednisolone on neuropathic pain and spinal glial activation in rats: anesthesiology.

[99] Tashiro S, Shinozaki M, Mukaino M, Renault-Mihara F, Toyama Y, Liu M, Nakamura M, Okano H (2015). BDNF induced by treadmill training contributes to the suppression of spasticity and allodynia after spinal cord injury via upregulation of KCC2. Neurorehabil Neural Repair.

[100] Tighilet B, Bordiga P, Cassel R, Chabbert C (2019). Peripheral vestibular plasticity vs central compensation: evidence and questions. J Neurol.

[101] Tighilet B, Brezun JM, Sylvie DD, G., Gaubert, C., Lacour, M.,  (2007). New neurons in the vestibular nuclei complex after unilateral vestibular neurectomy in the adult cat: Reactive neurogenesis in adult vestibular lesioned cats. Eur J Neurosci.

[102] Tighilet B, Chabbert C (2019). Adult neurogenesis promotes balance recovery after vestibular loss. Prog Neurobiol.

[103] Tighilet, B., Dutheil, S., Siponen, M.I., Noreña, A.J., 2016. Reactive neurogenesis and down-regulation of the potassium-chloride cotransporter KCC2 in the cochlear nuclei after cochlear deafferentation. Front. Pharmacol. 7. 10.3389/fphar.2016.00281 10.3389/fphar.2016.00281 PMC5005331 27630564

[104] Tighilet B, Leonard J, Bernard-Demanze L, Lacour M (2015). Comparative analysis of pharmacological treatments with N-acetyl-dl-leucine (Tanganil) and its two isomers (N-acetyl-L-leucine and N-acetyl-D-leucine) on vestibular compensation: Behavioral investigation in the cat. Eur J Pharmacol.

[105] Tighilet B, Leonard J, Lacour M (1995). Betahistine dihydrochloride treatment facilitates vestibular compensation in the cat. J Vestib Res.

[106] Tighilet B, Manrique C, Lacour M (2009). Stress axis plasticity during vestibular compensation in the adult cat. Neuroscience.

[107] Tighilet B, Péricat D, Frelat A, Cazals Y, Rastoldo G, Boyer F, Dumas O, Chabbert C (2017). Adjustment of the dynamic weight distribution as a sensitive parameter for diagnosis of postural alteration in a rodent model of vestibular deficit. PLoS ONE.

[108] Tighilet B, Trottier S, Mourre C, Lacour M (2006). Changes in the histaminergic system during vestibular compensation in the cat: histamine and vestibular compensation. J Physiol.

[109] Uffer DS, Hegemann SCA (2016). About the pathophysiology of acute unilateral vestibular deficit – vestibular neuritis (VN) or peripheral vestibulopathy (PVP)?. VES.

[110] Vidal, P.P., Wang, D.H., Graf, W., de Waele, C., 1993. Chapter 22 Vestibular control of skeletal geometry in the guinea pig: a problem of good trim?, in: Progress in Brain Research. Elsevier, pp. 229–243. 10.1016/S0079-6123(08)62282-7 10.1016/s0079-6123(08)62282-7 8234750

[111] Walker MF (2009). Treatment of vestibular neuritis. Curr Treat Options Neurol.

[112] Widera D, Mikenberg I, Elvers M, Kaltschmidt C, Kaltschmidt B (2006). Tumor necrosis factor α triggers proliferation of adult neural stem cells via IKK/NF-κB signaling. BMC Neurosci.

[113] Yamamoto T (2000). The Effect of Stress Application on Vestibular Compensation. Acta Otolaryngol.

[114] Yin Y, Sun W, Li Z, Zhang B, Cui H, Deng L, Xie P, Xiang J, Zou J (2013). Effects of combining methylprednisolone with rolipram on functional recovery in adult rats following spinal cord injury. Neurochem Int.

[115] Yoo MH, Yang CJ, Kim SA, Park MJ, Ahn JH, Chung JW, Park HJ (2017). Efficacy of steroid therapy based on symptomatic and functional improvement in patients with vestibular neuritis: a prospective randomized controlled trial. Eur Arch Otorhinolaryngol.

[116] Young LR, Bernard-Demanze L, Dumitrescu M, Magnan J, Borel L, Lacour M (2012). Postural performance of vestibular loss patients under increased postural threat. J Vestib Res.

